# The impact of equol-producing status in modifying the effect of soya isoflavones on risk factors for CHD: a systematic review of randomised controlled trials

**DOI:** 10.1017/jns.2016.18

**Published:** 2016-07-19

**Authors:** Rahel L. Birru, Vasudha Ahuja, Abhishek Vishnu, Rhobert W. Evans, Yoshihiro Miyamoto, Katsuyuki Miura, Takeshi Usui, Akira Sekikawa

**Affiliations:** 1 Department of Environmental and Occupational Health and Department of Epidemiology, University of Pittsburgh, Pittsburgh, PA, USA; 2 Health Sciences Center, West Virginia University, Morgantown, WV, USA; 3 Department of Preventive Cardiology, National Cerebral and Cardiovascular Center, Suita, Osaka, Japan; 4 Center for Epidemiologic Research in Asia, Shiga University of Medical Science, Seta-Tsukinowa-cho, Otsu, Shiga 520-2192, Japan; 5 Clinical Research Institute, National Hospital Organization, Kyoto Medical Center, Kyoto, Kyoto 612-8555, Japan

**Keywords:** Equol, Soya isoflavones, CHD, Risk factors, HDL-C, HDL-cholesterol, LDL-C, LDL-cholesterol, RCT, randomised controlled trial

## Abstract

Recent studies suggest that the ability to produce equol, a metabolite of the soya isoflavone daidzein, is beneficial to coronary health. Equol, generated by bacterial action on isoflavones in the human gut, is biologically more potent than dietary sources of isoflavones. Not all humans are equol producers. We investigated whether equol-producing status is favourably associated with risk factors for CHD following an intervention by dietary soya isoflavones. We systematically reviewed randomised controlled trials (RCT) that evaluated the effect of soya isoflavones on risk factors for CHD and that reported equol-producing status. We searched PubMed, EMBASE, Ovid Medline and the Cochrane Central Register for Controlled Trials published up to April 2015 and hand-searched bibliographies to identify the RCT. Characteristics of participants and outcomes measurements were extracted and qualitatively analysed. From a total of 1671 studies, we identified forty-two articles that satisfied our search criteria. The effects of equol on risk factors for CHD were mainly based on secondary analyses in these studies, thus with inadequate statistical power. Although fourteen out of the forty-two studies found that equol production after a soya isoflavone intervention significantly improved a range of risk factors including cholesterol and other lipids, inflammation and blood pressure variables, these results need further verification by sufficiently powered studies. The other twenty-eight studies primarily reported null results. RCT of equol, which has recently become available as a dietary supplement, on CHD and its risk factors are awaited.

CHD is the leading cause of morbidity and mortality in the USA^(^^1^^)^ and worldwide^(^^2^^)^. Nutrition is an important determinant for the risk of developing CHD; poor dietary habits are estimated to account for 20 % of CHD cases in the US adult population^(^^1^^)^. Soya foods are a potential nutritional source for modifying biomarkers of CHD^(^^3^^,^^4^^)^. One of the main components of soya that may exert protective cardioprotective effects are isoflavones, bioactive phyto-oestrogens found in soyabeans^(^^3^^)^. The predominant soya isoflavones are genistein, daidzein and glycitein. Isoflavones may reduce the risk of CHD by: (1) their action via oestrogen receptor β, due to their structural similarity to oestradiol, leading to decreased vasodilation and inflammation^(^^4^^–^^7^^)^; (2) their antioxidant activity, which may prevent the oxidative damage to LDL-cholesterol (LDL-C) that contributes to atherogenesis^(^^8^^)^; and (3) modulating the vascular system, reducing atherosclerotic lesions and improving vascular reactivity and vascular stiffness^(^^9^^,^^10^^)^.

Although there are clear cardiovascular benefits of isoflavones *in vitro* and in animal studies^(^^9^^,^^11^^)^, the evidence in humans is conflicting^(^^12^^–^^14^^)^. A growing hypothesis is that the ability of humans to metabolise daidzein to equol, referred to as ‘equol producers’, may contribute to the protective effects of soya^(^^15^^,^^16^^)^. Equol has a greater affinity for oestrogen receptors than its precursor daidzein^(^^17^^)^, a longer half-life and bioavailability in plasma than daidzein and genistein^(^^3^^,^^18^^)^, and more potent antioxidant activity than any other isoflavone^(^^3^^)^. Therefore, the potential beneficial effects of soya isoflavones for CHD and its risk factors may be greater among equol producers. While all tested animals, including rodents and monkeys, can produce equol, not all humans have the gut microflora required to convert daidzein to equol, a bioactive metabolite^(^^15^^,^^19^^)^.

Equol is a promising candidate for hindering the initiation and progression of atherosclerosis due to its ability to induce vasorelaxation and its anti-inflammatory and antioxidant activity^(^^20^^)^. Specifically, it induces vasorelaxation through enhancing the production of endothelium nitric oxide synthase-derived NO^(^^21^^)^. It can also inhibit NO derived by inducible nitric oxide synthase, expressed by immune cells during host defence, which is linked to atherosclerosis development^(^^22^^)^. Furthermore, equol prevents lipid and lipoprotein peroxidation, a crucial process in the pathogenesis of atherosclerosis^(^^23^^,^^24^^)^.

The purpose of the present review is to examine if there is a difference in the cardioprotective effect of soya isoflavones in humans based on the hosts’ ability to produce equol. No previous reviews have thoroughly examined the impact of equol-producing status on risk factors for CHD. We conducted a comprehensive search of the scientific literature to identify randomised controlled trials (RCT) that evaluated the effects of soya isoflavones on risk factors for CHD and selected studies that included analyses based on equol producer status.

## Methods

### Literature search

The systematic review was conducted following the Preferred Reporting Items for Systematic Reviews and Meta-Analyses (PRISMA) guidelines^(^^25^^)^. We initially searched PubMed (1950 to April 2015), EMBASE through Embase.com (1966 to April 2015), Ovid Medline (1946 to April 2015) and the Cochrane Library (Cochrane Central Register of Controlled Trials, 1999 to April 2015) for papers in any language using one or more textual or medical subject heading (MESH) terms for isoflavones (isoflavones, isoflavonoids, genistein, daidzein, equol), risk factors for CHD (cardiovascular disease, coronary heart disease, myocardial infarction, lipids, low-density lipoprotein-cholesterol, triglyceride, lipoproteins, hypercholesterolemia, lipid metabolism, blood pressure, glucose, vital signs, arterial stiffness, vascular stiffness, intima-media thickness, inflammation, endothelial function, endothelium, adipocytes) and RCT (randomised control study, clinical trial, placebo, intervention studies, pilot projects, sampling studies, twin studies, prospective studies, double blind study, single blind study, epidemiologic research design). We reviewed the reference lists of the collected articles to identify additional potentially relevant papers not identified by the original keyword search.

### Study selection

Studies were selected for the systematic review if they met the following criteria: (1) RCT; (2) full-text was published in English; (3) analysed adult subjects who ingested soya with isoflavones or isolated isoflavones as an intervention; (4) analysed traditional risk factors for CHD (including lipids, inflammatory, blood pressure, glycaemic and body composition variables) as outcome measurements; (5) determined the equol producer status of the participants; and (6) stratified the outcome measurements by equol producer status. The exclusion criteria included reviews or commentaries.

### Data synthesis and quality assessment

Searching, data extraction and the quality assessment were completed by two authors independently according to the inclusion criteria. Discrepancies were resolved by consensus. For each RCT, extracted data included sample size, baseline characteristics of the participants (sex, mean age, health status, demographics, equol producer status), study design, treatment regimen (dose, duration, isoflavone content, and type of soya intervention), and the assessment of the risk factor(s) for CHD.

The quality of the RCT methodology was graded using a fourteen-point evaluation tool for controlled clinical trials developed by the National Heart, Lung, and Blood Institute^(^^26^^)^. Questions were answered with a ‘yes’, ‘no’, ‘not reported’, ‘cannot determine’ or ‘not applicable’. The evaluation was based on the primary outcome measurements of the RCT. The RCT were given an overall rating of ‘good’, ‘fair’ or ‘poor’ at the discretion of the reviewers based on the guidelines provided by this tool.

## Results

### Search results

**Fig. 1.**
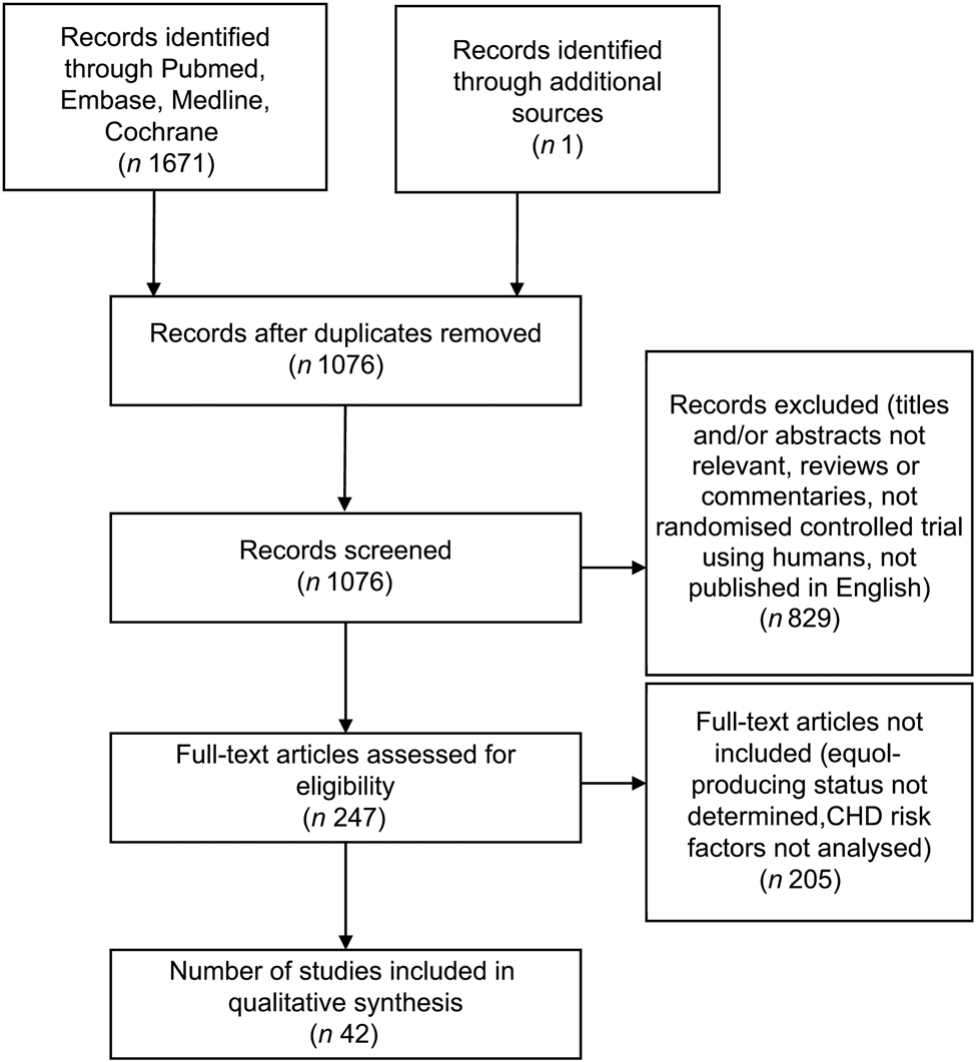
Study flow diagram of screened, excluded and analysed publications.

A total of 1671 papers were collected and, of these, 829 were excluded because they were not RCT, did not measure the traditional risk factors for CHD, or were not published in English (Fig. 1). Of the remaining 247 papers screened, forty-two met the selection criteria for this review. An outline of our search strategy using PubMed is provided in Supplementary Table S1.

### Study characteristics

Study characteristics are summarised in Tables 1 and 2, and Supplementary Table S2. Thirty studies included only female participants^(^^14^^,^^27^^–^^55^^)^, eleven studies included both males and females^(^^56^^–^^66^^)^, and one study had only male participants^(^^67^^)^. Of the forty-one studies involving women, thirty-four included postmenopausal women only^(^^14^^,^^27^^,^^28^^,^^30^^–^^55^^,^^60^^,^^63^^,^^65^^,^^66^^)^. The age of the participants ranged from 27 to 73 years. Participants were hypercholesterolaemic in seven studies^(^^56^^,^^57^^,^^59^^,^^62^^,^^64^^–^^66^^)^, hyperlipidaemic in two studies^(^^58^^,^^66^^)^, prehypertensive or hypertensive in five studies^(^^39^^–^^41^^,^^55^^,^^59^^)^, had type 2 diabetes in two studies^(^^30^^,^^61^^)^, had the metabolic syndrome in two studies^(^^27^^,^^63^^)^, and considered healthy in twenty-three studies^(^^14^^,^^29^^,^^31^^,^^34^^–^^38^^,^^42^^–^^54^^,^^60^^,^^67^^)^. Diet interventions in nineteen studies used soya protein isolate with isoflavone flour, or powder, or tablets^(^^31^^–^^33^^,^^37^^–^^42^^,^^48^^–^^51^^,^^57^^,^^58^^,^^61^^,^^62^^,^^65^^,^^67^^)^, fifteen used soya- and isoflavone-enriched milk or foods^(^^14^^,^^27^^,^^30^^–^^36^^,^^46^^,^^47^^,^^55^^–^^57^^,^^59^^,^^63^^–^^66^^)^, and nine used isolated isoflavone tablets or capsules^(^^28^^,^^29^^,^^42^^–^^45^^,^^52^^–^^54^^)^, with Gardner *et al.*^(^^57^^)^ using interventions that covered two categories. Isoflavone doses ranged from approximately 40 to 120 mg/d, with one dose particularly high at 900 mg/d^(^^44^^)^. Twenty-three studies examined cholesterol markers^(^^27^^,^^28^^,^^31^^,^^32^^,^^35^^,^^38^^,^^39^^,^^42^^,^^43^^,^^48^^,^^52^^,^^56^^–^^67^^)^, twenty-one examined other lipid variables^(^^27^^,^^28^^,^^31^^,^^32^^,^^35^^,^^38^^,^^39^^,^^42^^,^^43^^,^^47^^,^^48^^,^^52^^,^^56^^,^^58^^,^^59^^,^^61^^,^^62^^,^^64^^–^^67^^)^, eighteen examined blood pressure and vascular variables^(^^14^^,^^27^^,^^30^^,^^34^^–^^37^^,^^39^^,^^40^^,^^43^^,^^48^^,^^49^^,^^51^^,^^55^^,^^56^^,^^59^^,^^63^^,^^66^^)^, seventeen examined inflammatory markers^(^^27^^,^^33^^,^^34^^,^^42^^,^^44^^–^^46^^,^^48^^,^^50^^,^^53^^,^^54^^,^^56^^,^^62^^,^^63^^,^^65^^–^^67^^)^, ten examined glucose and insulin variables^(^^27^^,^^29^^,^^35^^,^^39^^,^^43^^,^^50^^,^^57^^,^^62^^,^^63^^,^^65^^)^ and five examined body composition variables^(^^27^^,^^41^^,^^43^^,^^65^^,^^66^^)^. There were numerous methods and standards used to distinguish equol producers from non-equol producers, including sampling from urine or serum, different threshold levels for differentiation, and various analytical techniques.

**Table 1.** Demographic and clinical characteristics of the participants in the randomised controlled trials (RCT) employing soya interventions and examining the effect of equol producer (EP) status on risk factors for CHD

First author, year	Country	Sex (no. M/no. F)	Mean age (years)	No. of EP (no. EP/no. NEP)	Guidelines for determining EP status	Subjects’ characteristics	Study design	Quality rating*
Acharjee *et al.* (2015)^(^^27^^)^†	USA	60 F	MetS: 54·1 (sd 6·5), without MetS: 54·6 (sd 5·8)	35 EP/25 NEP	Urinary equol concentration >1000 nmol/l	Postmenopausal, with and without MetS	CO	Fair
Badeau *et al.* (2007)^(^^28^^)^‡	Finland	30 F	54	15 EP/15 NEP	Equol concentration > five times baseline	Postmenopausal breast cancer survivors	CO, DB	Fair
Campbell *et al.* (2004)^(^^29^^)^	UK	23 F	Premenopausal: 34, postmenopausal: 57	7 EP/9 NEP in premenopausal group, 1 EP/6 NEP in postmenopausal group	Urinary equol concentrations >1 mg/ml	Healthy	CO, DB	Fair
Clerici *et al.* (2007)^(^^56^^)^	Italy	25 M/37 F	Control: 52·0 (sem 2·4), intervention: 58·1 (sem 2·2)	20 EP/9 NEP (of intervention group)	Plasma equol concentrations >83 nmol/l are EP, <40 nnmol/l are NEP, 24 h urinary log_10_ *S*-equol:daidzein ratio > −1·75 after daidzein challenge	Hypercholesterolaemic, adhering to Italian Heart Association Step II diet	CO, P, B	Fair
Curtis *et al.* (2013)^(^^30^^)^	UK	118 F	Control: 63·0 (sem 0·8), intervention: 62·1 (sem 0·7)	17 EP/30 NEP (of intervention group)	Not reported	Postmenopausal, type 2 diabetic, using statins	DB	Poor
Gallagher *et al.* (2004)^(^^31^^)^	USA	65 F	55	36 EP/29 NEP	Serum equol >10 ng/ml	Postmenopausal	DB	Poor
Gardner *et al.* (2007)^(^^57^^)^	USA	6 M/22 F	52 (sd 9)	9 EP/19 NEP	Plasma equol >50 nM	Hypercholesterolaemic	CO, SB	Poor
Greany *et al.* (2004)^(^^32^^)^§	USA	37 F	57·5 (sem 2·2)	8 EP/29 NEP	Plasma equol concentrations >15 nmol/l and urinary excretion >1500 nmol/24 h	Postmenopausal, history of breast cancer not treated with chemotherapy or no family history of breast cancer, no history of reproductive cancer	CO	Poor
Greany *et al.* (2008)^(^^33^^)^§	USA	34 F	57·7 (sd 6·0)	6 EP/28 NEP	Plasma equol concentration >15 nmol/l and urinary equol excretion >1500 nmol/d	Postmenopausal, with and without a history of breast cancer	CO	Poor
Hall *et al.* (2005)^(^^34^^)^‖	UK, Germany, Denmark, Italy	117 F	57·7 (sd 5·4)	33 EP/84 NEP	24 h urinary equol concentration during the isoflavone intervention >936 nmol/l	Postmenopausal	CO, DB	Fair
Hall *et al.* (2006)^(^^35^^)^‖	UK, Germany, Denmark, Italy	117 F	57·7 (sd 5·4)	33 EP/84 NEP	24 h urinary equol concentration during the isoflavone intervention >936 nmol/l	Postmenopausal	CO, DB	Fair
Hallund *et al.* (2006)^(^^36^^)^‖	Denmark, UK, Germany, Italy	28 F	57 (sd 5)	6 EP/22 NEP	24 h urinary equol concentration during the isoflavone intervention >936 nmol/l	Postmenopausal	CO, DB	Fair
Hodis *et al.* (2011)^(^^14^^)^	USA	350 F	60·9	39 consistent EP/35 intermittent EP/76 NEP	Consistent EP: plasma equol >20 nmol/l at all visits, intermittent EP: plasma equol >20 nmol/l at some visits, NEP: plasma equol never >20 nmol/l	Postmenopausal	DB	Good
Kreijkamp-Kaspers *et al.* (2005)^(^^37^^)^¶	Netherlands	175 F	Control: 66·8 (sd 4·7), intervention: 66·6	26 EP/62 NEP (of intervention group)	Plasma equol concentration >83 nmol/l	Postmenopausal	DB	Fair
Kreijkamp-Kaspers *et al.* (2004)^(^^38^^)^¶	Netherlands	175 F	Control: 66·7 (sd 4·8), intervention: 66·5 (sd 4·7)	26 EP/62 NEP (of intervention group)	Plasma equol concentration >83 nmol/l	Postmenopausal	DB	Fair
Liu *et al.* (2014)^(^^39^^)^**	China	287 F	Control: 58·5 (sd 4·7), whole soya: 57·6 (sd 5·3), daidzein: 57·7 (sd 5·0)	287 EP/0 NEP	24 h urinary log_10_ *S*-equol:daidzein ratio > −1·75 after daidzein challenge	Postmenopausal, prehypertensive	DB	Good
Liu *et al.* (2015)^(^^40^^)^**	China	265 F	Control: 58·5 (sd 4·7), whole soya: 57·6 (sd 5·3), daidzein: 57·7 (sd 5·0)	265 EP/0 NEP	24 h urinary log_10_ *S*-equol:daidzein ratio > −1·75 after daidzein challenge	Postmenopausal, prehypertensive or untreated hypertensive	P, DB	Good
Liu *et al.* (2013)^(^^41^^)^**	China	253 F	Control: 58·5 (sd 4·7), whole soya: 57·6 (sd 5·3), daidzein: 57·7 (sd 5·0)	253 EP/0 NEP	24 h urinary log_10_ *S*-equol:daidzein ratio > −1·75 after daidzein challenge	Postmenopausal, prehypertensive	DB	Good
Ma *et al.* (2005)^(^^58^^)^	USA	70 M/89 F	56 (sd 8·46)	21 EP/59 NEP (of intervention group)	Serum equol concentration >20 ng/ml	Hyperlipidaemic	DB	Fair
Mangano *et al.* (2013)^(^^42^^)^	USA	97 F	Control: 72·9 (sd 6·1), soya protein: 74·0 (sd 6·2), isoflavone: 72·3 (sd 5·7), soya protein and isoflavone: 73·0 (sd 5·7)	25 EP/26 NEP	12-month serum concentration of *S*-equol 20 nmol/l (5 µg/l)	Postmenopausal	DB	Poor
McVeigh *et al.* (2006)^(^^67^^)^	Canada	35 M	27·9 (sd 5·7)	12 EP/23 NEP	Urinary equol >1000 nmol/24 h	Healthy	CO, B	Poor
Meyer *et al.* (2004)^(^^59^^)^	Australia	13 M/10 F	54·0 (sem 1·8)	8 EP/15 NEP	Equol detected in the plasma or urine	Postmenopausal, hypercholesterolaemic and/or hypertensive	CO	Poor
Nestel *et al.* (2004^(^^60^^)^	Australia	46 M/34 F	Males: 58 (sd 7), Females: 58 (sd 6)	15 EP/65 NEP	Excretion of equol >1000 nmol/24 h	Postmenopausal	CO, P, DB	Fair
Nikander *et al.* (2004)^(^^43^^)^‡	Finland	56 F	54 (sd 6)	8 EP/40 NEP	EP: equol concentration >83 nmol/l, NEP: equol concentration <40 nmol/l	Postmenopausal	CO, DB	Fair
Pipe *et al.* (2009)^(^^61^^)^	Canada	16 M/13 F	60·1 (sd 9·64)	6 EP/23 NEP	Urinary equol > 1000 nmol/24 h	Postmenopausal, diet-controlled type 2 diabetic	CO, DB	Poor
Pop *et al.* (2008)^(^^44^^)^	USA	30 F	Placebo: 53·50 (se 1·06), intervention: 56·78 (se 1·25)	6 EP/23 NEP/1 intermediate EP	EP: plasma equol concentrations >20 µg/l; intermediate EP: (≥10 to ≤20 µg/l; NEP: plasma equol concentration <10 µg/l	Postmenopausal	DB	Poor
Pusparini & Hidayat (2015)^(^^45^^)^	Indonesia	182 F	Control EP: 54·3 (sd 3·42), control NEP: 52·2 (sd 3·24), intervention EP: 53·3 (sd 34·6), intervention NEP: 53·7 (sd 3·65)	110 EP/72 NEP	Baseline blood equol concentration >5 ng/ml	Postmenopausal	DB	Fair
Qin *et al.* (2014)^(^^62^^)^	China	91 M/86 F	Control: 52·9 (sd 6·0), low daidzein: 54·5 (sd 6·6), high daidzein: 53·4 (sd 6·4)	106 EP/71 NEP	Urinary equol concentration >1000 nmol/l, log_10_-transformed urinary *S*-equol:daidzein ratio > −1·75 after daidzein intervention	Hypercholesterolaemic	DB	Fair
Reimann *et al.* (2006)^(^^46^^)^‖	Denmark, UK, Germany	89 F	59 (sd 5)	29 EP/59 NEP	Urinary equol concentration >936 nmol/l urine	Postmenopausal	CO, DB	Poor
Reverri *et al.* (2015)^(^^63^^)^	USA	5 M/12 F	56 (sd 5)	8 EP/9 NEP	Equol/daidzein ≥0·018 with a daidzein threshold of ≥2 nmol/mg creatinine	Postmenopausal, MetS	CO	Poor
Sen *et al.* (2012)^(^^47^^)^	USA	82 F	39·2 (sd 6·1)	43 EP/39 NEP	Urinary daidzein excretion ≥2 nmol/mg creatinine, urinary equol:daidzein ≥0·018; participants who meet both criteria at least once during the study considered EP	Premenopausal	CO	Poor
Steinberg *et al.* (2003)^(^^48^^)^	USA	28 F	54·9 (sem 1·0)	10 EP/18 NEP	Not reported	Postmenopausal	CO, DB	Poor
Thorp *et al.* (2008)^(^^64^^)^	Australia	33 M/58 F	52·7 (sd 1·0)	30 EP/61 NEP	Urinary log_10_ *S-*equol:daidzein value > −1·75 after soya or daidzein intervention	Hypercholesterolaemic	CO, DB	Poor
Törmälä *et al.* (2008)^(^^49^^)^††	Finland	36 F	57·7 (sem 0·8)	16 EP/20 NEP	>4-fold rise in serum equol concentration	Postmenopausal, using tibolone	CO	Fair
Törmälä *et al.* (2008)^(^^50^^)^††	Finland	36 F	57·7 (sem 0·8)	16 EP/20 NEP	>4-fold rise in serum equol concentration	Postmenopausal, using tibolone	CO	Fair
Törmälä *et al.* (2007)^(^^51^^)^††	Finland	33 F	57·7 (sem 0·8)	14 EP/19 NEP	>4-fold rise in serum equol concentration	Postmenopausal, using tibolone	CO, DB	Fair
Törmälä *et al.* (2006)^(^^52^^)^‡	Finland	30 F	56 (sd 6)	15 EP/15 NEP	Equol concentration > five times baseline after soya isoflavone challenge	Postmenopausal, history of breast cancer	CO, DB	Fair
van der Velpen *et al.* (2014)^(^^53^^)^	Netherlands	Low genistein group (LG): 24 F; high genistein group (HG): 31 F	LG: 63·2 (sd 5·5); HG: 63·0 (sd 5·5)	LG: 7 EP/17 NEP; HG: 8 EP/23 NEP	Log_10_-transformed urinary *S*-equol:daidzein ratio > 1·75	Postmenopausal	CO, DB	Good
van der Velpen *et al.* (2013)^(^^54^^)^	Netherlands	30 F	61·1 (sd 5·8)	30 EP/0 NEP	Log_10_-transformed urinary *S*-equol:daidzein ratio > −1·75 post-isoflavone or daidzein challenge	Postmenopausal	CO, DB	Good
Welty *et al.* (2007)^(^^55^^)^†	USA	60 F	Normotensive: 53·5 (sd 5·3), hypertensive: 58·3 (sd 6·5)	35 EP/25 NEP	Urinary equol concentration greater than 1000 nmol/l	Postmenopausal; hypertensive, prehypertensive, or normotensive	CO	Fair
West *et al.* (2005)^(^^65^^)^	USA	14 M/18 F	Males: 57·36 (se 1·43), females using HRT: 57·17 (se 2·18), females not using HRT: 59·08 (se 1·54)	11 EP/21 NEP	High concentrations of equol in urine	Postmenopausal, hypercholesterolaemic, adhering to National Cholesterol Education Program Step I diet	CO, DB	Fair
Wong *et al.* (2012)^(^^66^^)^	Canada	42 M/43 F	59·9 (sd 8·9)	30 EP/55 NEP	Urinary equol >1000 nmol/24 h and log_10_-transformed urinary equol:daidzein ratio > −1·75	Postmenopausal, hypercholesterolaemic, hyperlipidaemic	Studies 1 and 2: CO; study 3: P	Poor

M, male; F, female; NEP, non-equol producer; MetS, metabolic syndrome; CO, crossover; DB, double-blind; P, parallel; B, blinded; SB, single-blinded; HRT, hormone replacement therapy.

* The quality of the RCT were evaluated based on the main outcomes reported. RCT were given a score of ‘good’, ‘fair’ or ‘poor’ after appraising the degree to which flaws in the study designs could affect the validity of the results.

† Studies that are or potentially using shared study participants.

‡ Studies that are or potentially using shared study participants.

§ Studies that are or potentially using shared study participants.

‖ Studies that are or potentially using shared study participants.

¶ Studies that are or potentially using shared study participants.

** Studies that are or potentially using shared study participants.

†† Studies that are or potentially using shared study participants.

**Table 2.** Characteristics of the soya isoflavone interventions used in the randomised controlled trials examining the effect of equol producer (EP) status on the risk factors for CHD

First author, year	Source of isoflavones	Control	Isoflavone dose/d	Constituents of isoflavone dose	Duration of trial
Acharjee *et al.* (2015)^(^^27^^)^*	TLC diet with soya nuts	TLC diet without soya nuts	101 mg isoflavones (AG)/d	30 mg daidzein, 61 mg genistein, 10 mg glycitein	16 weeks
Badeau *et al.* (2007)^(^^28^^)^†	Isoflavone tablet	Placebo tablet	114 mg isoflavones/d	41 mg daidzein, 7 mg genistein, 66 mg glycitein	6 months
Campbell *et al.* (2004)^(^^29^^)^	Isoflavone tablet	Placebo tablet	86 mg red clover isoflavones/d	43 mg total isoflavones: 4 mg genistein, 5 mg daidzein, 25 mg biochanin, 8 mg formononetin	2 months
Clerici *et al.* (2007)^(^^56^^)^	Soya germ-enriched pasta	Conventional pasta	33 mg of isoflavones (AG)/d	Predominantly daidzein, genistein, glycitein	8 weeks
Curtis *et al.* (2013)^(^^30^^)^	Flavonoid-enriched chocolate	Placebo chocolate	100 mg isoflavones (AG)/d	Predominantly daidzein	1 year
Gallagher *et al.* (2004)^(^^31^^)^	SPI powder with isoflavones (SPI 96 or SPI 50)	SPI without isoflavones (SPI 4)	SPI 96: 96 mg/d isoflavones, SPI 50: 52 mg isoflavones/d	SPI 96: 28 mg daidzein, 52 mg genistein; SPI 50: 20 mg daidzein, 28 mg genistein; SPI 4: 0 mg daidzein, 4 mg genistein	9 months (soya for 9 months, followed an additional 6 months)
Gardner *et al.* (2007)^(^^57^^)^	WB milk, SPI milk	Dairy milk	WB: 125 (sd 17) isoflavones (AG)/d; SPI milk: 39 (sd 1) isoflavones (AG)/d	WB milk: 56·4 (sd 6·4) mg daidzein, 65·5 (sd 9·7) mg genistein, 2·9 (sd 0·4) mg glycitein; SPI milk: 12·9 (sd 0·2) mg daidzein, 24·7 (sd 0·3) mg genistein, 1·0 (sd 0·2) mg glycitein	12 weeks
Greany *et al.* (2004)^(^^32^^)^‡	SPI, SPI plus probiotic capsules	MPI powder	44 (sem 8) mg isoflavones/d	34 % daidzein, 57 % genistein, 9 % glycitein	24 weeks
Greany *et al.* (2008)^(^^33^^)^‡	SPI powder	MPI powder	44 (sd 8) mg isoflavones/d	34 % daidzein, 57 % genistein, 9 % glycitein	24 weeks
Hall *et al.* (2005)^(^^34^^)^§	Isoflavone-enriched cereal bars	Placebo cereal bars	50 mg isoflavones/d	Genistein:daidzein ratio of 2	16 weeks
Hall *et al.* (2006)^(^^35^^)^§	Isoflavone-enriched cereal bars	Placebo cereal bars	50 mg isoflavones/d	Genistein:daidzein ratio of 2	16 weeks
Hallund *et al.* (2006)^(^^36^^)^§	Isoflavone-enriched cereal bars	Placebo cereal bars	50 mg isoflavones/d	Genistein:daidzein ratio of 2	16 weeks
Hodis *et al.* (2011)^(^^14^^)^	Powdered soya beverage or food bars	Total milk protein beverage or food bars	91 mg isoflavones/d (154 mg total isoflavone conjugates plus AGs)	36 mg AG daidzein 36 mg (61 mg total), 52 mg AG genistein (88 mg total), 3 mg AG glycitein (5 mg total)	2·5–3 years
Kreijkamp-Kaspers *et al.* (2005)^(^^37^^)^‖	Soya protein powder	Total milk protein powder	25·6 g of isoflavone-rich soya protein/d	41 mg daidzein, 52 mg genistein, 6 mg glycitein (AG) in 36·5 g soya-protein powder	12 months
Kreijkamp-Kaspers *et al.* (2004)^(^^38^^)^‖	Soya protein powder	Total milk protein powder	25·6 g of isoflavone-rich soya protein/d	41 mg daidzein, 52 mg genistein, 6 mg glycitein (AG) in 36·5 g soya-protein powder	12 months
Liu *et al.* (2014)^(^^39^^)^¶	Whole soya group: soya flour; daidzein group: daidzein and milk powder	Low-fat milk powder	Whole soya group: 40 g soya with 49·8 mg total isoflavones/d; daidzein group: 63 mg daidzein/d	Whole soya group: 23·2 mg daidzein, 19·4 mg genistein; daidzein group: 63 mg daidzein	6 months
Liu *et al.* (2015)^(^^40^^)^¶	Whole soya group: soya flour; daidzein group: daidzein and milk powder	Low-fat milk powder	Whole soya group: 49·3 mg isoflavones/d; daidzein group: 63 mg daidzein/d	Whole soya group: 23·2 mg daidzein, 19·4 mg genistein, 6·4 mg glycitein; daidzein group: 63 mg daidzein	6 months
Liu *et al.* (2013)^(^^41^^)^¶	Whole soya group: soya flour; daidzein group: daidzein and milk powder	Low-fat milk powder	Whole soya group: 40 g soya with 49·8 mg total isoflavones/d; daidzein group: 63 mg daidzein/d	Whole soya group: 23·2 mg daidzein, 19·4 mg genistein, 6·7 mg glycitein; daidzein group: 63 mg daidzein	6 months
Ma *et al*. (2005)^(^^58^^)^	Soya protein powder	Milk protein powder	120 mg isoflavones (AG)/d	Not reported	5 weeks
Mangano *et al.* (2013)^(^^42^^)^	SPI: soya protein and isoflavone tablets, SPP: soya protein and placebo tablets, CPI: control protein and isoflavone tablets	CPP: control protein and placebo tablets	Soya protein: 4 mg isoflavones/d; isoflavone tablets: 105 mg isoflavones (AG)/d	Primarily daidzein, genistein, glycitein and their β-glycosides	1 year
McVeigh *et al.* (2006)^(^^67^^)^	Low-Iso SPI: low-isoflavone SPI, high-Iso SPI: high-isoflavone SPI powders	MPI powder	Low-Iso SPI: 1·64 (sd 0·19) mg isoflavones (AG)/d; high-Iso SPI: 61·7 (sd 7·35) mg isoflavones (AG)/d	Not reported	171 d
Meyer *et al.* (2004)^(^^59^^)^	Soya milk, soya yogurt	Dairy milk, dairy yogurt	80 mg isoflavones/d	Soya milk: 8·8 mg isoflavones/100 g, soya yogurt: 8·8 mg isoflavones/100 g	10 weeks
Nestel *et al.* (2004)^(^^60^^)^	Red clover pill (B or F preparations)	Placebo pill	40 mg isoflavones/d of B or F preparations	Red clover B: <1 % daidzein, 4 % genistein, red clover F: <1 % daidzein and genistein	12 weeks
Nikander *et al.* (2004)^(^^43^^)^†	Isoflavonoid tablets	Placebo tablets	114 mg isoflavonoids/d	41 mg daidzein, 7 mg genistein, 66 mg glycitein	6 months
Pipe *et al.* (2009)^(^^61^^)^	SPI powder	MPI powder	88 mg isoflavones (AG)/d	27 mg daidzein, 57 mg genistein, 4 mg glycitein	114 d
Pop *et al.* (2008)^(^^44^^)^	Isoflavone capsules	Placebo capsule	900 mg isoflavones/d	296 mg daidzein, 558 mg genistein, 44 mg glycitein	84 d
Pusparini & Hidayat (2015)^(^^45^^)^	Soya isoflavone tablets	Placebo tablet	40 mg isoflavones/d	16·4 mg daidzein, 22·4 mg genistein, 1·2 mg glycitein	6 months
Qin *et al.* (2014)^(^^62^^)^	SPI with daidzein (DAI40 and DAI80) supplementation	SPI without daidzein supplementation	0·7 mg isoflavones/d supplemented with 40 mg/d daidzein (DAI40) or 80 mg/d daidzein (DAI80)	DAI40: 40 mg daidzein, DAI80: 80 mg daidzein	6 months
Reimann *et al.* (2006)^(^^46^^)^§	Isoflavone-enriched fruit cereal bars	Fruit cereal bar without isoflavones	50 mg isoflavones/d	Genistein:daidzein ratio of 2:1	16 weeks
Reverri *et al.* (2015)^(^^63^^)^	Soya nuts	Cookies supplemented with whey protein and fibre	101 mg isoflavones (AG)/d	42 mg daidzein, 55 mg genistein, 4 mg glycitein	8 weeks
Sen *et al.* (2012)^(^^47^^)^	High-soya group: two servings of soya foods/d; low-soya group: three servings of soya/week	None	High soya group: >40 mg of isoflavones/d; low soya group: <10 mg of isoflavones/d	Not reported	12 months
Steinberg *et al.* (2003)^(^^48^^)^	Soya+: SPI with isoflavones, soya-: SPI with trace amounts of isoflavones	Total milk protein	Soya+: 107·67 mg isoflavones/d (AG); soya−: 1·82 mg isoflavones/d (AG)	Soya+: 0·5 mg daidzein, 1 mg genistein, 0·5 mg glycitein (AG); soya-: 47 mg daidzein, 55 mg genistein, 5 mg glycitein (AG)	18 weeks
Thorp *et al.* (2008)^(^^64^^)^	Diet S: food with soya protein, diet SD: food with soya and dairy protein	Diet D: dairy protein	Diet S: 71·4 (sem 1·9) mg isoflavones (AG)/d; diet SD: 76 (sem 1·5) mg isoflavones (AG)/d; diet D: 0·5 (sem 0·1) mg isoflavones (AG)/d	Not reported	18 weeks
Törmälä *et al.* (2008)^(^^49^^)^**	Soya protein powder	Milk protein powder	112 mg isoflavones (AG)/d	Not reported	16 weeks
Törmälä *et al.* (2008)^(^^50^^)^**	Soya protein powder	Milk protein powder	112 mg isoflavones/d	43 mg daidzein, 63 mg genistein, 6 mg glycitein	16 weeks
Törmälä *et al.* (2007)^(^^51^^)^**	Soya protein powder	Milk protein powder	112 mg isoflavones/d	43 mg daidzein, 63 mg genistein, 6 mg glycitein	16 weeks
Törmälä *et al.* (2006)^(^^52^^)^†	Isoflavone tablet	Placebo tablet	114 mg isoflavones/d	41 mg daidzein, 7 mg genistein, 66 mg glycitein	6 months
van der Velpen *et al.* (2014)^(^^53^^)^	Isoflavone capsule	Placebo capsule	Low genistein (LG): 100 mg isoflavones (AG)/d; high genistein (HG): 104 mg isoflavones/d	LG: 56 mg daidzein and daidzin, 16 mg genistein and genistin, 28 mg glycitein and glycitin; HG: 51 mg daidzein and daidzin, 43 mg genistein and genistin, 10 mg glycitein and glycitin	16 weeks
van der Velpen *et al.* (2013)^(^^54^^)^	Isoflavone capsule	Placebo capsule	94 mg isoflavones (AG)/d	56 mg daidzein, 12 mg genistein, 26 mg glycitein	16 weeks
Welty *et al.* (2007)^(^^55^^)^*	TLC diet with soya nuts	TLC diet without soya nuts	101 mg isoflavones (AG)/d	30 mg daidzein, 61 mg genistein, 10 mg glycitein	16 weeks
West *et al.* (2005)^(^^65^^)^	SPI powder	MPI powder	90 mg isoflavones/d	Not reported	12 weeks
Wong *et al.* (2012)^(^^66^^)^	Soya food with isoflavones (three different diet protocols)	N/A	Low-isoflavone group: 10 mg/d; High-isoflavone group: 73 mg/d	Study 1: not reported; study 2: 28·4 mg daidzein, 29·7 mg genistein, 2·4 mg glycitein, study 3: not reported	4–8 weeks

TLC, therapeutic lifestyle changes; AG, aglycone; SPI, soya protein isolate; WB, whole bean soya; MPI, milk protein isolate; N/A, not applicable.

* Studies that are or potentially using shared study participants.

† Studies that are or potentially using shared study participants.

‡ Studies that are or potentially using shared study participants.

§ Studies that are or potentially using shared study participants.

‖ Studies that are or potentially using shared study participants.

¶ Studies that are or potentially using shared study participants.

** Studies that are or potentially using shared study participants.

### Synthesis of results

We categorised both the effects of soya isoflavones and equol producer status on the examined CHD risk factors as beneficial, negligible, or adverse (Tables 3–8). We analysed each risk factor independently; therefore the RCT were potentially categorised more than once. Twenty-two studies found statistically significant improvements in the risk factors for CHD after the soya isoflavone intervention compared with placebo. Of these, equol producer status further improved risk factors for CHD in six studies (including LDL-C, TAG, systolic blood pressure, diastolic blood pressure, flow-mediated dilation, soluble intercellular adhesion molecule-1, platelet-selectin and C-reactive protein). Equol producer status was comparable to the soya intervention in sixteen studies (including total cholesterol, LDL-C, HDL-cholesterol (HDL-C), TAG, apoB, systolic blood pressure, diastolic blood pressure, nitrate and nitrite, systemic arterial compliance, peak flow velocity, aortic augmentation index and IL-6).

**Table 3.** Randomised clinical trial results reporting the effect of soya isoflavone interventions and equol producer (EP) status on cholesterol and other lipid parameters*

First author, year	CHD risk factor measured	Effect of isoflavone on CHD risk factors	Result marker†	Effect of EP status on CHD risk factors	Result marker‡
Acharjee *et al.* (2015)^(^^27^^)^	TAG	Reduction in TAG in women with MetS (17·8 %, *P* = 0·04) after the soya intervention compared with placebo, unlike in women without MetS	+	Reduction in TAG in EP with MetS (22·9 %, *P* = 0·02) after the soya intervention compared with placebo. There were NS effects on NEP with or without MetS in TAG	+
Clerici *et al.* (2007)^(^^56^^)^	LDL-C, isoprostane excretion	Reduction in LDL-C (8·6 %, *P* = 0·002) compared with placebo after the soya intervention. Isoprostane excretion reduced from 58 (sem 6) ng/l at baseline to 39 (sem 4) ng/l after 4 weeks in the soya group (*P* < 0·001) (not measured in placebo group)	+	LDL-C reduced 15 (sem 7) mg/dl more in EP than in NEP (*P* = 0·042) after the soya intervention. Isoprostane excretion decreased in both EP and NEP, though more significantly in EP (*P* = 0·012) than NEP (*P* = 0·038)	+
Hall *et al.* (2006)^(^^35^^)^	%sdLDL-C	The isoflavone intervention was associated with a greater reduction of %sdLDL-C compared with placebo (24·14 (sd 14·26) and 22·22 (sd 11·87), respectively; *P* = 0·044)	+	The interaction between positive EP status and treatment was significant for %sdLDL-C (*P* < 0·05)	+
Hall *et al.* (2006)^(^^35^^)^	Lp(a)	The isoflavone intervention had a NS effect on Lp(a) compared with placebo	0	There was an interaction between EP status and treatment for Lp(a) (*P* < 0·05, data highly skewed)	+
Mangano *et al.* (2013)^(^^42^^)^	TC:HDL-C, LDL-C:HDL-C	The soya intervention had a NS effect on the risk factors compared with placebo	0	EP had lower TC:HDL and LDL-C:HDL-C compared with NEP (*P* = 0·018 and 0·043, respectively) after the isoflavone intervention	+
McVeigh *et al.* (2006)^(^^67^^)^	LDL-C	The soya intervention had a NS effect on LDL-C compared with placebo	0	EP status associated with a significant decrease in LDL-C on the low-isoflavone diet (*P* = 0·035) and high-isoflavone diet (*P* = 0·041) compared with placebo	+
Meyer *et al.* (2004)^(^^59^^)^	TC, LDL-C, LDL-C:HDL-C, TAG, Lp(a)	The soya intervention had a NS effect on the risk factors compared with placebo	0	EP status associated with significant reductions (*P* < 0·001) in TC (8·5 %), LDL-C (10 %), LDL-C:HDL-C ratio (13·5 %), TAG (21 %) and Lp(a) (11 %) after the soya intervention, unlike NEP	+
Pipe *et al.* (2009)^(^^61^^)^	TC, apoB	The isoflavone intervention had a NS effect on the risk factors compared with placebo	0	There was an interaction between EP status and TC (*P* = 0·05) and apoB (*P* = 0·04) after the soya intervention. There were NS effects of the soya intervention on TC or apoB in EP or NEP when analysed separately	+
Wong *et al.* (2012)^(^^66^^)^	HDL-C, apoA-I	The soya interventions had a NS effect on the risk factors compared with placebo	0	Apo A-I reduced in NEP but not EP (−0·08 (se 0·02) and −0·02 (se 0·02) g/l, respectively; *P* = 0·010) and HDL-C reduced in NEP but not EP (−0·07 (se 0·02) and 0·0 (se 0·03) mmol/l, respectively; *P* = 0·036) after the soya interventions	+
Badeau *et al.* (2007)^(^^28^^)^	Pre-(β) HDL-C	Pre-(β) HDL-C increased by 18 % (*P* < 0·05) after the isoflavone treatment	+	EP status had a NS effect on pre-(β) HDL-C levels after the isoflavone intervention	0
Clerici *et al.* (2007)^(^^56^^)^	TC	TC reduced after the soya intervention compared with placebo (7·3 %, *P* = 0·001)	+	TC reduction was greater in EP than NEP (*P* = 0·103) after the soya intervention	0
Gardner *et al.* (2007)^(^^57^^)^	LDL-C	LDL-C decreased after both soya interventions compared with placebo (161 (sd 20), 161 (sd 26), and 170 (sd 24) mg/dl for the WB soya milk, SPI milk, and dairy milk, respectively; *P* = 0·02 for each soya milk *v*. dairy milk)	+	EP status had a NS effect on LDL-C after either soya milk intervention	0
Greany *et al.* (2004)^(^^32^^)^	TC, LDL-C, HDL-C, TAG	Reductions in TC (−2·2 %, *P* = 0·02), LDL-C (−3·5 %, *P* = 0·006) and TAG (−8·8 %, *P* = 0·07) while HDL-C increased (4·2 %, *P* = 0·006) after the soya intervention compared with control	+	EP status had a NS effect on the risk factors on in all subjects, hypercholesterolaemic subjects alone, or normocholesterolaemic subjects alone after the soya intervention	0
McVeigh *et al.* (2006)^(^^67^^)^	TC:HDL-C, LDL-C:HDL-C, apoB:apoA-I	Reductions in TC:HDL-C, LDL-C:HDL-C, apoB:apoA-I after the soya diets (*P* = 0·031, 0·006, 0·011, respectively in the low-soya diet, *P* = 0·054, 0·012, 0·005, respectively in the high-soya diet) compared with control	+	Interaction of EP status and treatment was NS for the risk factors	0
Nestel *et al.* (2004)^(^^60^^)^	LDL-C	LDL-C reduced after the genistein-rich (biochanin) isoflavone intervention compared with placebo (*P* = 0·026)	+	EP status had a NS effect on LDL-C after the isoflavone interventions	0
Pipe *et al.* (2009)^(^^61^^)^	LDL-C, LDL-C:HDL-C, apoB:apoA-I	Reductions in LDL-C (*P* = 0·04), LDL-C:HDL-C (*P* = 0·04), and apoB:apoA-I (*P* = 0·05) after the isoflavone intervention compared with placebo	+	EP status had a NS effect on the risk factors after the isoflavone intervention	0
Qin *et al.* (2014)^(^^62^^)^	TAG	Reduction in the low- and high-daidzein interventions compared with placebo in TAG (−0·15 (sd 0·062) and 0·24 (sd 0·61) mmol/l, respectively; *P* < 0·05)	+	EP status had a NS effect on TAG after the isoflavone intervention	0
Thorp *et al.* (2008)^(^^64^^)^	TC, TAG	The soya diet caused a 3 % greater reduction in TC (−0·17 (sem 0·06) mmol/l, *P* < 0·05) and 4 % greater reduction in TAG (−0·14 (sem 0·05) mmol/l; *P* < 0·05) compared with control	+	NS interaction between EP status and diet treatment on the risk factors (*P* > 0·68 for all).	0
Wong *et al.* (2012)^(^^66^^)^	LDL-C, apoB	Reductions in LDL-C and apoB after the soya treatments compared with placebo (*P* values not provided)	+	EP status had a NS effect on the risk factors after the soya treatments	0
Acharjee *et al.* (2015)^(^^27^^)^	TC, LDL-C, HDL-C	The soya intervention had a NS effect on the risk factors compared with placebo	0	EP status had a NS effect on the risk factors compared with placebo	0
Badeau *et al.* (2007)^(^^28^^)^	ABCA1-dependent cholesterol efflux, TC, HDL-C, HDL-2, HDL-3, TC:HDL-C, non-HDL-C, TAG, apoA-I	The isoflavone intervention had a NS effect on the risk factors compared with placebo	0	EP status had a NS effect on the lipid risk factors. ABCA1-dependent cholesterol efflux values were higher in EP than NEP (3·4 (sd 1·4) % and 2·7 (sd 0·6) %, respectively), though NS, after the isoflavone intervention	0
Gallagher *et al.* (2004)^(^^31^^)^	TC, LDL-C, HDL-C, TAG, apoA-I, apoB	The soya intervention had a NS effect on the risk factors compared with placebo	0	NS differences in percentage change between equol levels and the risk factors after the isoflavone intervention	0
Hall *et al.* (2006)^(^^35^^)^	TC, LDL-C, HDL-C, TAG, TC:HDL-C	The isoflavone intervention had a NS effect on the risk factors compared with placebo	0	EP status had a NS effect on the risk factors after the isoflavone intervention	0
Kreijkamp-Kaspers *et al.* (2004)^(^^38^^)^	TC, HDL-C, LDL-C, TAG, Lp(a)	The soya intervention had a NS effect on the risk factors compared with placebo	0	NS interaction with EP status and any of the risk factors	0
Ma *et al*. (2005)^(^^58^^)^	TC, HDL-C, LDL-C, TAG	The isoflavone intervention had a NS effect on the risk factors compared with placebo	0	EP status had a NS effect on the risk factors after the isoflavone intervention	0
Mangano *et al.* (2013)^(^^42^^)^	TC, HDL-C, LDL-C, TAG	The soya intervention had a NS effect on the risk factors compared with placebo	0	EP status had a NS effect on the risk factors after the soya intervention	0
McVeigh *et al.* (2006)^(^^67^^)^	TC, HDL-C, non-HDL-C, TAG, apoA-I, apoB	The soya intervention had a NS effect on the risk factors compared with placebo	0	NS interaction with EP status and the soya intervention and any of the risk factors	0
Meyer *et al.* (2004)^(^^59^^)^	HDL-C	The soya intervention had a NS effect on HDL-C compared with placebo	0	EP status had a NS effect on HDL-C after the soya intervention	0
Nestel *et al.* (2004)^(^^60^^)^	LDL-C	The intervention of isoflavones isolated from red clover enriched in formononetin had a NS effect on LDL-C compared with placebo	0	EP status had a NS effect on LDL-C after the isoflavone treatments	0
Nikander *et al.* (2004)^(^^43^^)^	TC, LDL-C, HDL-C, TAG, apoA-I, apoB, Lp(a)	The isoflavonoid intervention had a NS effect on the risk factors compared with placebo though in women with baseline levels of LDL-C above the median LDL-C, it increased (*P* = 0·009)	0	EP status had a NS effect on the risk factors after the isoflavonoid intervention	0
Pipe *et al.* (2009)^(^^61^^)^	HDL-C, non-HDL-C, TAG, apoA-I, TC: HDL-C, TAG:HDL-C, non-HDL:HDL-C	The isoflavone intervention had a NS effect on the risk factors compared with placebo	0	EP status had a NS effect on the risk factors after the isoflavone intervention	0
Qin *et al.* (2014)^(^^62^^)^	HDL-C, LDL-C, apoA-I, apoB, Lp(a)	The isoflavone intervention had a NS effect on the risk factors compared with placebo	0	EP status had a NS effect on the risk factors after the isoflavone intervention	0
Reverri *et al.* (2015)^(^^63^^)^	OxLDL-C	The soya intervention had a NS effect on oxLDL-C compared with placebo	0	EP status had a NS effect on oxLDL-C after the soya intervention	0
Steinberg *et al.* (2003)^(^^48^^)^	TC, LDL-C, HDL-C, TC:HDL-C, TAG, CD formation	The isoflavone intervention had a NS effect on the risk factors	0	EP status had a NS effect on the risk factors after the soya interventions	0
Thorp *et al.* (2008)^(^^64^^)^	LDL-C, HDL-C, TC:HDL-C	The soya intervention had a NS effect on the risk factors	0	NS interaction between EP status and diet treatment on the risk factors after the soya intervention (*P* > 0·68 for all)	0
Törmälä *et al.* (2006)^(^^52^^)^	TC, HDL-C, LDL-C, TAG, apoA-I, apoB, serum cholesterol efflux capacity	The isoflavone intervention had a NS effect on the risk factors compared with placebo	0	EP status had a NS effect on the risk factors after the isoflavone intervention	0
West *et al.* (2005)^(^^65^^)^	TC, HDL-C, LDL-C, apoA-I, apoB, Lp(a)	The soya intervention had a NS effect on the risk factors compared with placebo	0	EP status had a NS effect on the risk factors after the soya intervention	0
Wong *et al.* (2012)^(^^66^^)^	TC, TC:HDL-C, LDL-C:HDL-C, TAG, apoB:apoA-I	The soya interventions had a NS effect on the risk factors on the risk factors compared with placebo	0	EP status had a NS effect on the risk factors after the soya treatments	0
Sen *et al.* (2012)^(^^47^^)^	Isoprostane excretion	There was a positive association between isoprostane excretion and isoflavones after the high soya diet intervention (*P* = 0·02)	–	There was a positive association between isoprostane excretion and the isoflavone intervention for EP (*P* = 0·03) but not NEP (*P* = 0·32) after the high-soya diet intervention	–

MetS, metabolic syndrome; NEP, non-equol producer; LDL-C, LDL-cholesterol; sdLDL-C, small dense LDL-C; Lp, lipoprotein; TC, total cholesterol; HDL-C, HDL-cholesterol; WB, whole bean soya; SPI, soya protein isolate; ABCA1, adenosine triphosphate-binding cassette A1; CD, conjugated diene formation; OxLDL-C, oxidised LDL-C.

* Results are first stratified by the impact of EP status and then the impact of the soya isoflavone interventions on each of the lipid risk factors.

† +, Beneficial effect of soya isoflavones on risk factors of CHD; 0, negligible effect of soya isoflavones on risk factors of CHD; –, adverse effect of soya isoflavones on risk factors of CHD.

‡ +, Beneficial effect of EP status on risk factors of CHD after soya intervention; 0, negligible effect of EP status on CHD risk factors after soya intervention; –, adverse effect of EP status on risk factors of CHD after soya intervention.

**Table 4.** Randomised clinical trial results reporting the effect of soya isoflavone interventions and equol producer (EP) status on blood pressure and vasculature parameters*

First author, year	CHD risk factor measured	Effect of isoflavone on CHD risk factors	Result marker†	Effect of EP status on CHD risk factors	Result marker‡
Acharjee *et al.* (2015)^(^^27^^)^	DBP	Reduction in DBP in women with and without MetS (5·4 %, *P* = 0·03 and 3·4 %, *P* = 0·0008, respectively) after the soya intervention	+	EP with and without MetS had reduced DBP (7·7 %, *P* = 0·02 and 3·3 %, *P* = 0·02, respectively) after the soya intervention compared with placebo. There were NS effects on NEP with or without MetS in DBP	+
Clerici *et al.* (2007)^(^^56^^)^	FMD	Increase in FMD (2 (sem 0·8) %; *P* = 0·012) after the soya intervention compared with placebo	+	Increase in FMD in EP from baseline concentrations (*P* = 0·03) after the soya intervention, unlike in NEP	+
Welty *et al.* (2007)^(^^55^^)^	SBP	Reduction in SBP in hypertensive women (9·9 %, *P* = 0·003) and normotensive women (5·2 %, *P* *<* 0·001) after the soya intervention compared with the placebo	+	In the 8 of 12 hypertensive women with LDL-C levels greater than 140 mg/dl (>3·63 mmol/l), the percentage reduction in SBP was positively correlated with the level of equol in the soya diet arm (*r* 0·80; *P* = 0·02)	+
Curtis *et al.* (2013)^(^^30^^)^	BP, DBP, MAP, PWV	The flavonoid intervention had a NS effect on BP and PWV compared with placebo. The flavonoid intervention had a NS greater reduction compared with placebo in DBP (*P* = 0·06) and MAP (*P* = 0·06)	0	EP compared with NEP had reduced BP (*P* = 0·01), DBP (EP: −2·24 (se 1·31) mmHg; NEP: 1·00 (se 0·89) mmHg; *P* < 0·01), MAP (EP: −1·24 (se 1·30) mmHg; NEP: 1·90 (se 1·08) mm Hg; *P* = 0·01) and PWV (EP: −0·68 (se 0·40) m/s; NEP: 0·32 (se 0·55) m/s; *P* = 0·001). In EP, an inverse correlation between DBP and urinary equol concentrations was observed (*r* −0·44, *P* = 0·08)	+
Acharjee *et al.* (2015)^(^^27^^)^	SBP	Reduction of SBP in women with and without MetS (5·9 %, *P* < 0·001 and 6·7 %, *P* = 0·01, respectively) after the soya intervention compared with placebo	+	SBP changed in both EP (6·4 %, *P* < 0·001) and NEP (5·4 %, *P* = 0·003) in women without MetS compared with placebo. In women with MetS, NS change in SBP in EP or NEP	0
Hallund *et al.* (2006)^(^^36^^)^	NMD, NOx, NOx:ET-1, SAC	Reductions in NMD (15·5 % *v*. 12·4 %, *P* = 0·03), NOx (*P* = 0·003), NOx:ET-1 (*P* = 0·005) and SAC (*P* = 0·04) after the soya intervention compared with placebo	+	NS interaction between EP status and vascular responses to isoflavones and placebo treatment	0
Reverri *et al.* (2015)^(^^63^^)^	AIx	Reduction in AIx after the soya intervention compared with placebo (*P* = 0·03)	+	EP status had a NS effect on AIx after the soya intervention	0
Steinberg *et al.* (2003)^(^^48^^)^	PFV	Reduction in PFV after the soya intervention compared with placebo (37 %; *P* = 0·03)	+	EP status had a NS effect on PFV after the soya intervention	0
Welty *et al.* (2007)^(^^55^^)^	DBP	Reduction in DBP after the soya intervention in hypertensive women (6·8 % mmHg, *P* = 0·001) and normotensive women (2·9 %; *P* = 0·02) compared with the placebo	+	EP status had a NS effect on DBP after the soya intervention	0
Wong *et al.* (2012)^(^^66^^)^	SBP, DBP	Reductions in DBP and SBP after the soya treatments compared with placebo (*P* values not provided)	+	EP status had a NS effect on the risk factors after the soya treatments	0
Curtis *et al.* (2013)^(^^30^^)^	SBP, total plasma NO concentrations, ET-1	The flavonoid intervention had a NS effect on the risk factors compared with placebo. There was a NS greater decrease in SBP the flavonoid group compared with placebo (*P* = 0·07)	0	EP status had a NS effect on the risk factors after the flavonoid intervention	0
Hall *et al.* (2005)^(^^34^^)^	BP, ET-1, vWF	The isoflavone intervention had a NS effect on the risk factors compared with placebo	0	EP status had a NS effect on the risk factors after the isoflavone intervention	0
Hall *et al.* (2006)^(^^35^^)^	Mean SBP, Mean DBP	The isoflavone intervention had a NS effect on the risk factors compared with placebo	0	EP status had a NS effect on the risk factors after the isoflavone intervention	0
Hallund *et al.* (2006)^(^^36^^)^	FMD, ET-1, BP, IAC, arterial volume, arterial distensibility, SVR	The isoflavone intervention had a NS effect on the risk factors compared with placebo. There was a NS greater increase in SVR after the isoflavone intervention compared with placebo (*P* = 0·06)	0	NS interaction between EP status and the risk factors after the isoflavone intervention	0
Hodis *et al.* (2011)^(^^14^^)^	CIMT	There was a NS greater reduction in CIMT progression after the isoflavone intervention compared with control (16 %; *P* = 0·36)	0	EP status had a NS effect on CIMT progression rate after the isoflavone intervention	0
Kreijkamp-Kaspers *et al.* (2005)^(^^37^^)^	DBP, %FMD	The soya intervention had a NS effect on the risk factors compared with placebo	0	EP status had a NS effect on the risk factors after the soya intervention	0
Meyer *et al.* (2004)^(^^59^^)^	HDL-C, MAP, SBP, DBP, arterial compliance	The soya intervention had a NS effect on the risk factors compared with placebo	0	EP status had a NS effect on the risk factors after the soya intervention	0
Nikander *et al.* (2004)^(^^43^^)^	BP	The isoflavonoid intervention had a NS effect on the risk factors compared with placebo	0	EP status had a NS effect on the risk factors after the isoflavonoid intervention	0
Pusparini & Hidayat (2015)^(^^45^^)^	NO	The isoflavone intervention had a NS effect on NO compared with placebo	0	EP status had a NS effect on NO after the isoflavone intervention	0
Reverri *et al.* (2015)^(^^63^^)^	Reactive hyperemia index	The soya intervention had a NS effect on the risk factor compared with placebo	0	EP status had a NS effect on the risk factor after the soya intervention	0
Steinberg *et al.* (2003)^(^^48^^)^	Brachial artery vessel diameter, ET-1, total NO	The soya interventions had a NS effect on the risk factors	0	EP status had a NS effect on the risk factors after the soya interventions	0
Törmälä *et al.* (2008)^(^^49^^)^	AIx, EFI	The soya intervention had a NS effect on the risk factors compared with placebo	0	EP status had a NS effect on the risk factors after the soya intervention. EP taking tibolone had lower AIx (*P* = 0·01) and EPI (*P* = 0·009) compared with NEP	0
Törmälä *et al.* (2007)^(^^51^^)^	SBP, DBP, MAP	The soya intervention had a NS effect on the risk factors compared with placebo	0	EP status had a NS effect on the risk factors after the soya intervention. EP women taking tibolone had lower SBP (*P* = 0·02), DBP (*P* = 0·01) and MAP (*P* = 0·007) which was maintained after the soya intervention	0
Kreijkamp-Kaspers *et al.* (2005)^(^^37^^)^	SBP	Increase in SBP after the soya intervention compared with placebo (4·3 mmHg; *P* = 0·04)	–	EP status had a NS effect on the risk factors after the soya intervention	0

DBP, diastolic blood pressure; MetS, metabolic syndrome; NEP, non-equol producer; FMD, flow-mediated dilation; SBP, systolic blood pressure; LDL-C, LDL-cholesterol; BP, blood pressure; MAP, mean arterial pressure; PWV, carotid to femoral pulse wave velocity; NMD, nitroglycerine-mediated endothelium-independent vasodilation; NOx, nitrate and nitrite; ET-1, endothelin-1; SAC, systemic arterial compliance; AIx, augmentation index; PFV, peak flow velocity; vWF, von Willebrand factor; IAC, isobaric arterial compliance; SVR, systemic vascular resistance; CIMT, carotid artery intima-media thickness; HDL-C, HDL-cholesterol; EFI, endothelial function index.

* Results are first stratified by the impact of EP status and then the impact of the soya isoflavone interventions on each of the lipid risk factors.

† +, Beneficial effect of soya isoflavones on risk factors of CHD; 0, negligible effect of soya isoflavones on risk factors of CHD; –, adverse effect of soya isoflavones on risk factors of CHD.

‡ +, Beneficial effect of EP status on risk factors of CHD after soya intervention; 0, negligible effect of EP status on CHD risk factors after soya intervention; –, adverse effect of EP status on risk factors of CHD after soya intervention.

**Table 5.** Randomised clinical trial results reporting the effect of soya isoflavone interventions and equol producer (EP) status on inflammation and DNA damage parameters*

First author, year	CHD risk factor measured	Effect of isoflavone on CHD risk factors	Result marker†	Effect of EP status on CHD risk factors	Result marker‡
Acharjee *et al.* (2015)^(^^27^^)^	CRP, sICAM-1	Reduction in CRP in women with and without MetS (11·8 %, *P* = 0·04 and 30 %, *P* = 0·01, respectively) after the soya intervention compared with placebo. In women with MetS, reduction in sICAM-1 (5·2 %, *P* = 0·04) compared with placebo, unlike in women without MetS	+	Reduced CRP (21·4 %; *P* = 0·01) and sICAM-1 (7·3 %, *P* = 0·03) in EP with MetS compared with placebo after the soya intervention. Reduced CRP (30 %; *P* = 0·04) in EP without MetS compared with placebo. There were NS effects on NEP with or without MetS in any of these variables	+
Clerici *et al.* (2007)^(^^56^^)^	hsCRP	Reduction in hsCRP (2·2 (sem 0·9) mg/l, *P* = 0·03) after the soya intervention compared with placebo	+	After the soya intervention, hsCRP decreased 0·9 (sem 0·5) mg/l more in EP than NEP (*P* = 0·025)	+
Pusparini & Hidayat (2015)^(^^45^^)^	MDA	Reduction in MDA after the soya intervention (*P* = 0·021)	+	After the soya intervention, EP had a greater decline in MDA than NEP	+
Törmälä *et al.* (2008)^(^^50^^)^	P-selectin	P-selectin decreased by 10·3 % (*P* = 0·002) after the soya intervention compared with placebo	+	EP had a greater decline in P-selectin (13·5 %; *P* = 0·007) than NEP (7·7 %; NS) after the soya intervention	+
Mangano *et al.* (2013)^(^^42^^)^	IL-6	The percentage change of IL-6 declined from baseline after the soya intervention compared with placebo (*P* = 0·007)	+	EP status had a NS effect on percentage change of IL-6 after the soya intervention	0
Qin *et al.* (2014)^(^^62^^)^	Uric acid	Reductions in the low and high daidzein isoflavone interventions compared with placebo in uric acid (−23 (sd 47) and −29 (sd 0·44) µmol/l, respectively; *P* < 0·05)	+	EP status had a NS effect on uric acid after the isoflavone intervention	0
Greany *et al.* (2008)^(^^33^^)^	Hcy, CRP, E-selectin, VCAM-1, ICAM-1	The soya intervention had a NS effect on the risk factors compared with placebo	0	EP status had a NS effect on the risk factors after the soya intervention	0
Hall *et al.* (2005)^(^^34^^)^	MCP-1, CRP, VCAM-1, ICAM-1, E-selectin	The isoflavone intervention had a NS effect on the risk factors compared with placebo	0	EP status had a NS effect on the risk factors after the isoflavone intervention	0
Mangano *et al.* (2013)^(^^42^^)^	hsCRP	The soya intervention had a NS effect on hsCRP compared with placebo	0	EP status had a NS effect on hsCRP after the soya intervention	0
McVeigh *et al.* (2006)^(^^67^^)^	CRP	The soya intervention had a NS effect on CRP compared with placebo	0	NS interaction with EP status and the soya intervention with CRP	0
Pop *et al.* (2008)^(^^44^^)^	Neutrophil count, DNA damage markers (AP-site assay, comet assay), apoptosis markers (TUNEL assay, caspase-3 activation)	The isoflavone intervention had a NS effect on the risk factors compared with placebo	0	EP status had a NS effect on the risk factors after the isoflavone intervention. Activated caspase-3 was higher in treated EP on day 1 but decreased through day 84, while it increased in NEP in this time period	0
Pusparini & Hidayat (2015)^(^^45^^)^	VCAM-1	The soya intervention had a NS effect on VCAM-1 compared with placebo	0	EP status had a NS effect on VCAM-1 after the soya intervention	0
Reimann *et al.* (2006)^(^^46^^)^	Hcy, ADMA	The isoflavone intervention had a NS effect on the risk factors compared with placebo	0	EP status had a NS effect on the risk factors after the isoflavone intervention	0
Reverri *et al.* (2015)^(^^63^^)^	CRP, TNF, IL-6, IL-18, IL-10	The soya intervention had a NS effect on the risk factors compared with placebo	0	EP status had a NS effect on the risk factors after the soya intervention	0
Steinberg *et al.* (2003)^(^^48^^)^	VCAM-1, ICAM-1, E-selectin	The soya intervention had a NS effect on the risk factors	0	EP status had a NS effect on the risk factors after the soya intervention	0
Törmälä *et al.* (2008)^(^^50^^)^	CRP, ICAM-1, VCAM-1	The soya intervention had a NS effect on the risk factors compared with placebo. There was a NS increase in VCAM-1 after the soya intervention compared with placebo (9·2 %; *P* = 0·06)	0	EP status had a NS effect on the risk factors after the soya intervention	0
West *et al.* (2005)^(^^65^^)^	VCAM-1, P-selectin	The soya intervention had a NS effect on the risk factors compared with placebo	0	EP status had a NS effect on the risk factors after the soya intervention	0
Wong *et al.* (2012)^(^^66^^)^	CRP	The soya interventions had a NS effect on CRP compared with placebo	0	EP status had a NS effect on CRP after the soya interventions	0

CRP, C-reactive protein; sICAM-1, soluble intercellular adhesion molecule-1; MetS, metabolic syndrome; NEP, non-equol producer; hsCRP, high-sensitivity C-reactive protein; MDA, malondialdehyde; P-selectin, platelet selectin; Hcy, homocysteine; E-selectin, endothelial selectin; VCAM-1, vascular cell adhesion molecule 1; ICAM-1, intracellular adhesion molecule-1; MCP-1, monocyte chemoattractant protein-1; AP-site, apurinic/apyrimidinic site; TUNEL, terminal deoxynucleotidyl transferase dUTP nick end labelling; ADMA, asymmetric dimethylarginine.

* Results are first stratified by the impact of EP status and then the impact of the soya isoflavone interventions on each of the lipid risk factors.

† +, Beneficial effect of soya isoflavones on risk factors of CHD; 0, negligible effect of soya isoflavones on risk factors of CHD; –, adverse effect of soya isoflavones on risk factors of CHD.

‡ +, Beneficial effect of EP status on risk factors of CHD after soya intervention; 0, negligible effect of EP status on CHD risk factors after soya intervention; –, adverse effect of EP status on risk factors of CHD after soya intervention.

**Table 6.** Randomised controlled trial results reporting the effect of soya isoflavone interventions and equol producer (EP) status on glucose and insulin parameters*

First author, year	CHD risk factor measured	Effect of isoflavone on CHD risk factors	Result marker†	Effect of EP status on CHD risk factors	Result marker‡
Acharjee *et al.* (2015)^(^^27^^)^	Glucose	The soya intervention had a NS effect on glucose compared with placebo	0	EP status had a NS effect on glucose compared with placebo	0
Campbell *et al.* (2004)^(^^29^^)^	IGF-1, IGF-BP1	The isoflavone intervention had a NS effect on the risk factors compared with placebo	0	EP status had a NS effect on the risk factors after the isoflavone intervention	0
Gardner *et al.* (2007)^(^^57^^)^	Glucose, insulin	The soya intervention had a NS effect on the risk factors compared with placebo	0	EP status had a NS effect on the risk factors after the soya intervention	0
Hall *et al.* (2006)^(^^35^^)^	Glucose, insulin	The isoflavone intervention had a NS effect on the risk factors compared with placebo	0	EP status had a NS effect on the risk factors after the isoflavone intervention	0
Nikander *et al.* (2004)^(^^43^^)^	Glucose, insulin	The isoflavonoid intervention had a NS effect on the risk factors compared with placebo	0	EP status had a NS effect on the risk factors after the isoflavonoid intervention	0
Qin *et al.* (2014)^(^^62^^)^	Glucose, insulin, glycated Hb	The isoflavone intervention had a NS effect on the risk factors compared with placebo	0	EP status had a NS effect on the risk factors after the isoflavone intervention	0
Reverri *et al.* (2015)^(^^63^^)^	Insulin	The soya intervention had a NS effect on insulin compared with placebo	0	EP status had a NS effect on insulin after the soya intervention	0
Törmälä *et al.* (2008)^(^^50^^)^	SHBG	The soya intervention had a NS effect on SHBG compared with placebo	0	EP status had a NS effect on SHBG after the soya intervention	0
West *et al.* (2005)^(^^65^^)^	Glucose	The soya intervention had a NS effect on glucose compared with placebo	0	EP status had a NS effect on glucose after the soya intervention	0
Reverri *et al.* (2015)^(^^63^^)^	Glucose	Glucose decreased after both snack interventions but decreased more after the control compared with the soya intervention (*P* = 0·02)	–	EP status had a NS effect on the risk factor after the soya intervention	0
Campbell *et al.* (2004)^(^^29^^)^	IGF-BP3	The isoflavone intervention had a NS effect on IGF-BP3 compared with placebo	0	Equol excretion was positively associated with IGF-BP3 concentrations in postmenopausal women at the end of the placebo phase (*r* 0·895; *P* = 0·04) and isoflavone intervention (*r* 0·984; *P* = 0·002)	–

IGF, insulin-like growth factor; IGF-BP1, insulin-like growth factor binding protein-1; SHBG, sex hormone binding globulin; IGF-BP3, insulin-like growth factor binding protein-3.

* Results are first stratified by the impact of EP status and then the impact of the soya isoflavone interventions on each of the lipid risk factors.

† +, Beneficial effect of soya isoflavones on risk factors of CHD; 0, negligible effect of soya isoflavones on risk factors of CHD; –, adverse effect of soya isoflavones on risk factors of CHD.

‡ +, Beneficial effect of EP status on risk factors of CHD after soya intervention; 0, negligible effect of EP status on CHD risk factors after soya intervention; –, adverse effect of EP status on risk factors of CHD after soya intervention.

**Table 7.** Randomised controlled trial results reporting the effect of soya isoflavone interventions and equol producer (EP) status on body composition variables*

First author, year	CHD risk factor measured	Effect of isoflavones on CHD risk factors	Result marker†	Effect of EP status on CHD risk factors	Result marker‡
Acharjee *et al.* (2015)^(^^27^^)^	BMI	The soya intervention had a NS effect on BMI compared with placebo	0	EP status had a NS effect on BMI compared with placebo	0
Nikander *et al.* (2004)^(^^43^^)^	BW	The isoflavonoid intervention had a NS effect on BW compared with placebo	0	EP status had a NS effect on BW after the isoflavonoid intervention	0
West *et al.* (2005)^(^^65^^)^	BW	The soya intervention had a NS effect on BW compared with placebo	0	EP status had a NS effect on BW after the soya intervention	0
Wong *et al.* (2012)^(^^66^^)^	BW, BMI, waist circumference	The soya interventions had a NS effect on the risk factors compared with placebo	0	EP status had a NS effect on the risk factors after the soya treatments	0

BW, body weight.

* Results are first stratified by the impact of EP status and then the impact of the soya isoflavone interventions on each of the lipid risk factors.

† +, Beneficial effect of soya isoflavones on risk factors of CHD; 0, negligible effect of soya isoflavones on risk factors of CHD; –, adverse effect of soya isoflavones on risk factors of CHD.

‡ +, Beneficial effect of EP status on risk factors of CHD after soya intervention; 0, negligible effect of EP status on CHD risk factors after soya intervention; –, adverse effect of EP status on risk factors of CHD after soya intervention.

**Table 8.** Results of the randomised clinical trials examining the effect of soya isoflavone interventions on the risk factors for CHD in equol producers (EP) only

First author, year	CHD risk factor measured	Effect of EP status on CHD risk factors	Result marker*
Liu *et al.* (2014)^(^^39^^)^	LDL-C, LDL-C:HDL-C, hsCRP, TAG, TC, HDL-C, glucose, NEFA, CIMT	Reductions from baseline after the whole soya intervention in LDL-C (−0·25 mmol/l; 95 % CI −0·19, −0·014), LDL-C:HDL-C (0·157; 95 % CI −0·318, 0·004) and hsCRP (−0·054 mg/l; 95 % CI −0·199, 0·012) compared with placebo. TAG were reduced at 6 months in the whole soya group compared with placebo (*P* < 0·05)	+
		The soya intervention had a NS effect on TC, HDL-C, glucose, NEFA and CIMT compared with placebo. The daidzein intervention had a NS effect on the risk factors compared with placebo	0
Liu *et al.* (2015)^(^^40^^)^	24 h, daytime, and night time DBP, SBP, MAP, %FMD	The soya and daidzein interventions had a NS effect on the risk factors compared with placebo	0
Liu *et al.* (2013)^(^^41^^)^	BW, BMI, waist and hip circumferences, waist:hip ratio, body fat percentage, fat mass, free-fat mass	The soya and daidzein interventions had a NS effect on the risk factors compared with placebo	0
Van der Velpen *et al.* (2014)^(^^53^^)^	Expression of inflammatory genes	Expression of inflammatory-related genes in the adipose tissue was up-regulated in EP and down-regulated in NEP in both isoflavone interventions. Further analysis identified a predominance of anti-inflammatory gene expression in EP	0
van der Velpen *et al.* (2013)^(^^54^^)^	Expression of inflammatory genes	The expression of 357 genes on a gene chip encoding 19 738 gene identifiers (1·8 %) significantly changed after isoflavone intervention in peripheral blood mononuclear cells of EP. There was a down-regulation of gene sets related to inflammation, driven by reduced TLR4, TIRAP and IL-1β gene expression and complement and coagulation gene sets	+

LDL-C, LDL-cholesterol; HDL-C, HDL-cholesterol; hsCRP, high-sensitivity C-reactive protein; TC, total cholesterol; CIMT, carotid artery intima-media thickness; DBP, diastolic blood pressure; SBP, systolic blood pressure; MAP, mean arterial pressure; FMD, flow-mediated dilation; BW, body weight; NEP, non-equol producers; TLR4, Toll-like receptor 4; TIRAP, toll–interleukin 1 receptor domain-containing adaptor protein.

* +, Beneficial effect of EP status on risk factors of CHD after soya intervention; 0, negligible effect of EP status on CHD risk factors after soya intervention; –, adverse effect of EP status on risk factors of CHD after soya intervention.

Forty studies found no association between soya isoflavones and risk factors for CHD compared with placebo. Of these, equol producer status significantly improved risk factors for CHD in seven studies (including total cholesterol, LDL-C, TAG, apoA-I, apoB, lipoprotein (a), blood pressure, diastolic blood pressure, mean arterial pressure, carotid to femoral pulse wave velocity). As with the soya isoflavone intervention, equol producer status was insignificant in thirty-two studies and was adverse in one study.

Three studies found that soya isoflavones had a negative effect on the risk factors of CHD. Of these, equol producer status was negligible in two studies and magnified the adverse outcomes of the soya isoflavone intervention in one study (isoprostane excretion). Equol producer status was also associated with the adverse outcome of an increase in insulin-like growth factor binding protein-3.

Five studies were comprised of participants who were all equol producers (Table 8); two of the studies found statistically significant beneficial effects of the isoflavone interventions on risk factors of CHD (including LDL-C, high-sensitivity C-reactive protein, TAG, inflammatory gene expression) while four studies observed negligible effects.

The RCT varied in quality, with the overall scores provided in Table 1 and the ratings summarised in Supplementary Table S3. Failure to report sample size calculations, details on the randomisation and allocation concealment procedures, and lack of intention-to-treat analyses or other suitable statistical method of dealing with participant drop-out were the most frequent flaws. Six RCT were given a ‘good’ rating, twenty were given a ‘fair’ rating and sixteen were given a ‘poor’ rating.

The heterogeneity of the studies in terms of populations, treatment regimens, intended duration, and outcomes prevented us from quantitatively synthesising the evidence in the form of a meta-analysis. Besides, the total number of participants included in all forty-two of the studies together was 3796, which, along with varying interventions and populations, probably provides insufficient statistical power to quantitatively measure the effect of dietary interventions. Further, most of these forty-two studies were small and had fewer than fifty participants, and only eighteen out of the forty-two studies qualified to be ‘fair’ or ‘good’ quality. The six ‘good’-quality papers (Hodis *et al.*^(^^14^^)^; Liu *et al.*^(^^39^^–^^41^^)^; van der Velpen *et al.*^(^^53^^,^^54^^)^) come from three different trials – while the Hodis study examined carotid artery intima-media thickness progression among equol producers and non-producers, Liu *et al.* and van der Velpen *et al.* examined their intervention only among equol producers. Liu *et al.* examined the effect of soya on risk factors such as lipid markers^(^^39^^–^^41^^)^, while van der Velpen *et al.*^(^^53^^,^^54^^)^ examined the effect of soya on the expression of inflammatory genes. Given these varying outcomes, we have chosen to not perform a meta-analysis in our present review.

## Discussion

Though the overall effect of equol producer status during a dietary soya intervention on risk factors of CHD is inconclusive, we found evidence of a favourable effect of equol producer status in fourteen of the forty-two studies^(^^27^^,^^30^^,^^35^^,^^39^^,^^42^^,^^45^^,^^50^^,^^54^^–^^56^^,^^59^^,^^61^^,^^66^^,^^67^^)^ regardless of the success of the soya intervention. Equol production was associated with positive changes in cholesterol^(^^35^^,^^39^^,^^42^^,^^56^^,^^59^^,^^61^^,^^66^^,^^67^^)^ and other lipid variables^(^^27^^,^^35^^,^^39^^,^^56^^,^^59^^,^^61^^,^^66^^)^, blood pressure measurements^(^^27^^,^^30^^,^^55^^,^^56^^)^ and inflammatory markers^(^^27^^,^^39^^,^^45^^,^^50^^,^^54^^,^^56^^)^. The effect of equol producer status was insignificant on CHD risk factors in forty studies^(^^14^^,^^27^^–^^46^^,^^48^^–^^67^^)^ and adverse in two studies^(^^29^^,^^47^^)^. We did not find consistent evidence of equol production affecting specific risk factors for CHD. The heterogeneity of the CHD risk factors analysed, sample size, study designs and quality, and definition of equol producers prevented quantitative synthesis of the results.

The majority of the studies in the present review retrospectively categorised study participants by equol producer status and conducted a secondary analysis of the effect of equol on the risk factors for CHD. Therefore, these RCT were very unlikely to be sufficiently powered to detect a difference in CHD risk factors between equol producers and non-equol producers. We identified ten studies with study designs that included enrolment criteria based on equol producer status^(^^28^^,^^39^^–^^41^^,^^49^^–^^54^^)^. Of these, three found equol producer status improved several CHD risk factors (LDL-C, LDL-C:HDL-C, TAG, platelet-selectin and inflammatory gene expression) after the soya intervention^(^^39^^,^^50^^,^^54^^)^ while the remaining associations measured in the RCT were negligible.

There are numerous differences in the experimental design of the RCT that could explain the inconsistency in the outcomes. The isoflavone dose ranged in both quantity and consistency between RCT. In particular, the amount of daidzein in the intervention formulations, which indicates the magnitude of equol that could be metabolised from daidzein and bioavailable in equol producers, largely varied between studies. Additionally, the duration and frequency of exposure to the intervention were inconsistent. Curtis *et al.*^(^^30^^)^ found that improvements in blood pressure, mean arterial pressure, and pulse wave velocity measures in equol producers were seen after 1 year but not at 6 months, suggesting that long-term exposure to isoflavones may be more beneficial.

The criteria used to define equol producers differed across the RCT included in our review, with variability in the biological samples used to measure equol, the concentration cut-offs selected to distinguish equol producers from non-equol producers, and the analytical methods used to measure equol. Setchell & Cole^(^^68^^)^ proposed classifying equol producers by a threshold log_10_-transformed ratio of *S*-(-)equol, a diastereoisomer of equol produced by the intestinal bacteria in humans, to its precursor daidzein of −1·75 in urine after a 3 d soya isoflavone challenge. This accounts for inconsistency in the technical measurements of equol and avoids classifying equol producers based on absolute measurements of equol, which exhibit greater variability^(^^68^^)^. Nine studies used this approach^(^^39^^–^^54^^,^^56^^,^^62^^,^^64^^,^^66^^)^, with four finding a beneficial effect of equol producer status on risk factors of CHD^(^^39^^,^^54^^,^^56^^,^^66^^)^ and eight finding a negligible effect^(^^39^^–^^41^^,^^53^^,^^56^^,^^62^^,^^64^^,^^66^^)^.

Further complicating the interpretation of the data are the potential sex differences in the metabolism of soya^(^^69^^)^, which could affect the bioavailability of isoflavone metabolites between males and females. In a meta-analysis examining the effects of soya isoflavones on lipids, subjects with hypercholesterolaemia had greater reductions in men than in women^(^^12^^)^. While there were studies of mixed sex (*n* 11) or of only males (*n* 1), the present review consisted primarily of female-only RCT, which may have masked the effects of equol producer status on the outcome measurements. Nestel *et al.*^(^^60^^)^ found that LDL-C was significantly reduced after supplementation with biochanin (a precursor of genistein) compared with placebo (*P* = 0·026); when results were stratified based on sex, males showed a significant reduction in median LDL-C levels of 9·5 % while females had no measurable difference. Equol producer status did not further reduce LDL-C, which the authors speculated was due to the small sample size of fifteen equol producers, with seven included in the biochanin intervention group^(^^60^^)^.

The source of soya may also contribute to the variability in its effectiveness. The type of processing used for soya products during production can affect the isoflavone content^(^^13^^)^ and modify other components of soya^(^^70^^)^. Additionally, soya protein isolate primarily contains isoflavone glucosides while fermented soya foods contain isoflavones mainly in the aglycone form^(^^15^^,^^71^^)^. Isoflavone aglycones are absorbed more efficiently than isoflavone glucosides in humans and may therefore be more effective in CHD prevention^(^^72^^)^. Daidzein in the aglycone form is also more readily converted to equol^(^^15^^)^. Clerici *et al.*^(^^56^^)^ found that pasta enriched in isoflavone aglycones significantly reduced total cholesterol, LDL-C, high-sensitivity C-reactive protein, and arterial stiffness compared with placebo in study participants, with effects more pronounced in equol producers. Of the fourteen RCT that found a positive association between equol producer status and CHD risk factors, seven used interventions of foods and milk enriched with soya^(^^27^^,^^30^^,^^35^^,^^55^^,^^56^^,^^59^^,^^66^^)^.

Furthermore, baseline age and the health status of the participants may contribute to variability in the outcome measurements. Oestrogen receptor β has been found to be enhanced in extracted arteries from postmenopausal CHD patients compared with normal subjects, with enhanced dilation in response to isoflavones^(^^73^^)^. Hodis *et al.* found that isoflavone supplementation failed to prevent the progression of subclinical atherosclerosis in healthy postmenopausal women overall; a subanalysis indicated, however, that healthy women within 5 years of becoming postmenopausal had a significantly reduced mean carotid artery intima-media thickness progression rate of 68 % compared with placebo^(^^74^^)^. Previous meta-analyses have also found lipid variables to be more positively affected by soya interventions in hypercholesterolaemic patients than in healthy subjects^(^^12^^,^^75^^)^. We identified thirty-five RCT that only used postmenopausal women; all of the studies that found a favourable association of equol producer status on risk factors of CHD had postmenopausal participants. There were a relatively equal number of RCT using healthy participants (*n* 20) *v*. participants with underlying health issues or a history of illness (*n* 22); of the fourteen studies that found equol producer status to improve risk factors for CHD, five had healthy participants^(^^35^^,^^42^^,^^45^^,^^54^^,^^67^^)^ while nine had participants with underlying health issues related to CHD^(^^27^^,^^30^^,^^39^^,^^50^^,^^55^^,^^56^^,^^59^^,^^61^^,^^66^^)^.

In the present systematic review, electronic databases were extensively searched following our defined set of guidelines and used to extract relevant data. Our results may imply that equol is beneficial on cardiovascular health, yet the interpretation is limited largely because of the secondary analysis of equol producers in RCT of dietary sources of isoflavones. Recently, equol itself has become available as a dietary supplement. Orally administered equol has greater plasma accumulation than other dietary sources of isoflavones^(^^76^^)^ and has the potential for enhanced therapeutic effects due to its more potent antioxidant properties and bioactivity among all isoflavones. In fact, one RCT of equol on risk factors of CHD has been conducted. Usui *et al.*^(^^77^^)^ found a statistically significant improvement in LDL-C, glycated HbA1c levels, and cardio-ankle vascular index scores, a measure of vascular stiffness, in overweight and obese patients after dietary equol supplementation, particularly for non-equol producers. This study is limited by its small sample size and short duration of the intervention. We recommend additional RCT of equol itself as an intervention to directly assess its effects on CHD risk factors and potentially CHD.

## Supplementary material

For supplementary material accompanying this paper visit http://dx.doi.org/10.1017/jns.2016.18. click here to view supplementary material
