# Supplementary material for: The impact of equol-producing status in modifying the effect of soya isoflavones on risk factors for CHD: a systematic review of randomised controlled trials
Source: J Nutr Sci. 2016 Jul 19;5:e30. doi: 10.1017/jns.2016.18 (PMC4976117; doi:10.1017/jns.2016.18)
Supplement: Supplementary file 1 [file S2048679016000185sup001.doc]

**Online Supplementary material**

**Supplementary Table S1.** Search in PubMed database

| Search | Query |
| --- | --- |
| [#26](http://www.ncbi.nlm.nih.gov/pubmed) | Search **(#17 AND #25)** |
| [#25](http://www.ncbi.nlm.nih.gov/pubmed) | Search **(#18 OR #19 OR #20 OR #21 OR #22 OR #23 OR #24)** |
| [#24](http://www.ncbi.nlm.nih.gov/pubmed) | Search **epidemiological studies** |
| [#23](http://www.ncbi.nlm.nih.gov/pubmed) | Search **intervention** |
| [#22](http://www.ncbi.nlm.nih.gov/pubmed) | Search **case control** |
| [#21](http://www.ncbi.nlm.nih.gov/pubmed) | Search **placebo** |
| [#20](http://www.ncbi.nlm.nih.gov/pubmed) | Search **trial** |
| [#19](http://www.ncbi.nlm.nih.gov/pubmed) | Search **randomized** |
| [#18](http://www.ncbi.nlm.nih.gov/pubmed) | Search **human** |
| [#17](http://www.ncbi.nlm.nih.gov/pubmed) | Search **(#5 AND #16)** |
| [#16](http://www.ncbi.nlm.nih.gov/pubmed) | Search **(#6 OR #7 OR #8 OR #9 OR #10 OR #11 OR #12 OR #13 OR #14 OR #15)** |
| [#15](http://www.ncbi.nlm.nih.gov/pubmed) | Search **triglyceride** |
| [#14](http://www.ncbi.nlm.nih.gov/pubmed) | Search **blood pressure** |
| [#13](http://www.ncbi.nlm.nih.gov/pubmed) | Search **LDL** |
| [#12](http://www.ncbi.nlm.nih.gov/pubmed) | Search **cardiovascular** |
| [#11](http://www.ncbi.nlm.nih.gov/pubmed) | Search **inflammation** |
| [#10](http://www.ncbi.nlm.nih.gov/pubmed) | Search **hypercholesterolemia** |
| [#9](http://www.ncbi.nlm.nih.gov/pubmed) | Search **endothelial function** |
| [#8](http://www.ncbi.nlm.nih.gov/pubmed) | Search **myocardial infarction** |
| [#7](http://www.ncbi.nlm.nih.gov/pubmed) | Search **lipids** |
| [#6](http://www.ncbi.nlm.nih.gov/pubmed) | Search **coronary** |
| [#5](http://www.ncbi.nlm.nih.gov/pubmed) | Search **(#1 OR #2 OR #3 OR #4)** |
| [#4](http://www.ncbi.nlm.nih.gov/pubmed) | Search **genistein** |
| [#3](http://www.ncbi.nlm.nih.gov/pubmed) | Search **daidzein** |
| [#2](http://www.ncbi.nlm.nih.gov/pubmed) | Search **equol** |
| [#1](http://www.ncbi.nlm.nih.gov/pubmed) | Search **isoflavone** |

**Supplementary Table S2.** Summary of the number of RCTs sharing particular characteristics

| Descriptor | # of RCTs |
| --- | --- |
| RCT Category |  |
| Randomized crossover design  Parallel | 29  4 |
| Gender |  |
| All female  All male  Mixed gender | 30  1  11 |
| Location |  |
| Asia  Australia  Europe  USA and Canada | 5  3  17  16 |
| Health Status |  |
| Healthy  Hypercholesterolemic  Hyperlipidemic  Prehypertensive/Hypertensive  Type II diabetes  Metabolic syndrome | 23  7  2  5  2  2 |
| Diet Intervention |  |
| Soy protein isolate with isoflavones  Soy or isoflavone enriched foods  Isoflavone Isolate | 19  15  9 |
| Categories of Result Markers Analyzed |  |
| Cholesterol and other lipids  Blood Pressure and vascular  Inflammatory  Glucose and insulin  Body composition | 23  21  17  10  5 |

**Supplementary Table S3.** Quality assessment of the RCTs

| First Author, Year | Randomized? | Method of randomization adequate? | Treatment allocation concealed? | Participants and providers blinded to the treatment? | People assessing the outcomes blinded to the participants' treatment? | Groups similar at baseline? | Drop-out rate 20% or lower? | Differential drop-out rate 15% or lower? | High adherence to the intervention? | Other interventions avoided or similar in the groups? | Outcomes assessed using valid measures, used for all participants? | Report that the sample size was sufficiently large (at least 80% power)? | Outcomes reported or subgroups analyzed prespecified? | Intention-to-treat analysis? | Quality Rating |
| --- | --- | --- | --- | --- | --- | --- | --- | --- | --- | --- | --- | --- | --- | --- | --- |
| Acharjee, 2015**^†^** ^(1)^ | Yes | NR | No | No | NR | Yes | Yes | Yes | Yes | Yes | Yes | No | No | Yes | Fair |
| Badeau, 2007**^‡^** ^(2)^ | Yes | Yes | Yes | Yes | Yes | Yes | Yes | NA | Yes | Yes | CD | NR | No | No | Fair |
| Campbell, 2004 ^(3)^ | Yes | NR | NR | Yes | Yes | No | NR | NR | Yes | Yes | Yes | Yes | Yes | NR | Fair |
| Clerici, 2007 ^(4)^ | Yes | NR | NR | Yes | NR | Yes | Yes | Yes | Yes | Yes | Yes | Yes | Yes | Yes | Fair |
| Curtis, 2013 ^(5)^ | Yes | Yes | NR | Yes | Yes | Yes | No | Yes | Yes | Yes | Yes | No | Yes | No | Poor |
| Gallagher, 2004 ^(6)^ | Yes | NR | NR | Yes | Yes | No | No | NR | Yes | Yes | Yes | Yes | Yes | No | Poor |
| Gardner, 2007 ^(7)^ | Yes | Yes | Yes | No | Yes | NR | Yes | NR | Yes | Yes | Yes | No | Yes | No | Poor |
| Greany, 2004^§^ ^(8)^ | Yes | NR | NR | NR | NR | NR | Yes | NR | Yes | Yes | Yes | No | Yes | No | Poor |
| Greany, 2008^§ (9)^ | Yes | NR | NR | NR | NR | No | NA | NA | Yes | Yes | Yes | No | No | No | Poor |
| Hall, 2005^ǁ^ ^(10)^ | Yes | NR | NR | Yes | Yes | Yes | Yes | NR | Yes | Yes | Yes | Yes | Yes | No | Fair |
| Hall, 2006^ǁ^ ^(11)^ | Yes | NR | NR | Yes | Yes | Yes | Yes | NR | Yes | Yes | Yes | Yes | Yes | No | Fair |
| Hallund, 2006^ǁ^ ^(12)^ | Yes | NR | NR | Yes | Yes | Yes | Yes | Yes | Yes | Yes | Yes | Yes | Yes | No | Fair |
| Hodis, 2011 ^(13)^ | Yes | Yes | Yes | Yes | Yes | Yes | Yes | Yes | Yes | Yes | Yes | Yes | Yes | Yes | Good |
| Kreijkamp-Kaspers, 2005^¶^ ^(14)^ | Yes | Yes | Yes | Yes | Yes | Yes | No | Yes | Yes | No | Yes | Yes | Yes | Yes | Fair |
| Kreijkamp-Kaspers, 2004^¶^ ^(15)^ | Yes | Yes | Yes | Yes | Yes | Yes | No | Yes | Yes | No | Yes | Yes | Yes | Yes | Fair |
| Liu, 2014^**^ ^(16)^ | Yes | Yes | Yes | Yes | Yes | Yes | Yes | Yes | Yes | Yes | Yes | Yes | Yes | Yes | Good |
| Liu, 2015^**^ ^(17)^ | Yes | Yes | Yes | Yes | Yes | Yes | Yes | Yes | Yes | Yes | Yes | Yes | Yes | Yes | Good |
| Liu, 2013^**^ ^(18)^ | Yes | Yes | Yes | Yes | Yes | Yes | Yes | Yes | Yes | Yes | Yes | Yes | Yes | Yes | Good |
| Ma, 2005 ^(19)^ | Yes | NR | NR | Yes | Yes | Yes | Yes | Yes | Yes | Yes | Yes | No | Yes | Yes | Fair |
| Mangano, 2013 ^(20)^ | Yes | NR | NR | Yes | Yes | No | No | CD | Yes | Yes | Yes | No | Yes | No | Poor |
| McVeigh, 2006 ^(21)^ | Yes | NR | NR | Yes | NR | NR | Yes | NR | Yes | Yes | Yes | No | Yes | No | Poor |
| Meyer, 2004 ^(22)^ | Yes | NR | NR | No | No | NR | CD | CD | Yes | Yes | Yes | Yes | Yes | CD | Poor |
| Nestel, 2004 ^(23)^ | Yes | Yes | NR | Yes | Yes | NR | Yes | Yes | CD | Yes | Yes | NR | Yes | N/A | Fair |
| Nikander, 2004**^‡^** ^(24)^ | Yes | Yes | NR | Yes | Yes | Yes | Yes | Yes | Yes | Yes | Yes | Yes | Yes | No | Fair |
| Pipe, 2009 ^(25)^ | Yes | NR | NR | Yes | Yes | NR | Yes | NR | NR | Yes | Yes | No | Yes | No | Poor |
| Pop, 2008 ^(26)^ | Yes | NR | NR | Yes | Yes | Yes | Yes | NR | NR | Yes | Yes | Yes | Yes | No | Poor |
| Pusparini, 2015 ^(27)^ | Yes | Yes | Yes | Yes | Yes | Yes | Yes | Yes | Yes | Yes | Yes | Yes | Yes | No | Fair |
| Qin, 2014 ^(28)^ | Yes | Yes | Yes | Yes | Yes | Yes | Yes | Yes | Yes | Yes | Yes | Yes | Yes | No | Fair |
| Reimann, 2006^ǁ^ ^(29)^ | Yes | NR | NR | Yes | Yes | Yes | Yes | NR | NR | Yes | Yes | No | Yes | No | Poor |
| Reverri, 2015 ^(30)^ | Yes | NR | NR | NR | NR | NR | Yes | Yes | Yes | Yes | Yes | Yes | Yes | No | Poor |
| Sen, 2012 ^(31)^ | Yes | NR | NR | No | NR | No | Yes | NR | Yes | Yes | Yes | No | Yes | No | Poor |
| Steinberg, 2003 ^(32)^ | Yes | NR | NR | Yes | Yes | Yes | No | NR | Yes | Yes | Yes | NR | Yes | No | Poor |
| Thorp, 2008 ^(33)^ | Yes | Yes | NR | Yes | Yes | NR | No | NR | Yes | Yes | Yes | No | Yes | No | Poor |
| Tormala, 2008**^††^** ^(34)^ | Yes | NR | NR | Yes | Yes | Yes | Yes | Yes | Yes | Yes | Yes | No | Yes | No | Fair |
| Tormala, 2008**^††^** ^(35)^ | Yes | NR | NR | Yes | Yes | Yes | Yes | Yes | Yes | Yes | Yes | No | Yes | No | Fair |
| Tormala, 2007**^††^** ^(36)^ | Yes | NR | NR | Yes | Yes | No | Yes | Yes | Yes | Yes | Yes | No | Yes | No | Fair |
| Tormala, 2006**^‡^** ^(37)^ | Yes | Yes | Yes | Yes | Yes | Yes | Yes | NA | Yes | Yes | Yes | No | No | No | Fair |
| van der Velpen, 2014 ^(38)^ | Yes | Yes | Yes | Yes | Yes | Yes | Yes | Yes | Yes | Yes | Yes | Yes | Yes | CD | Good |
| van der Velpen, 2013 ^(39)^ | Yes | Yes | Yes | Yes | Yes | Yes | Yes | Yes | Yes | Yes | Yes | Yes | Yes | CD | Good |
| Welty, 2007**^†^** ^(40)^ | Yes | NR | NR | NR | NR | Yes | Yes | Yes | Yes | Yes | Yes | No | Yes | Yes | Fair |
| West, 2005 ^(41)^ | Yes | NR | NR | Yes | Yes | Yes | Yes | NR | Yes | Yes | Yes | No | Yes | No | Fair |
| Wong, 2012 ^(42)^ | Yes | NR | No | No | No | Yes | Yes | Yes | Yes | No | No | No | No | No | Poor |

**^†^**, **^‡^**^, §, ǁ, ¶, **^,**^††^**Studies that are or potentially using shared study participants

References:

1. Acharjee S, Zhou JR, Elajami TK *et al.* (2015) Effect of soy nuts and equol status on blood pressure, lipids and inflammation in postmenopausal women stratified by metabolic syndrome status. *Metabolism: clinical and experimental* **64**, 236-243.

2. Badeau R, Jauhiainen M, Metso J *et al.* (2007) Effect of isolated isoflavone supplementation on ABCA1-dependent cholesterol efflux potential in postmenopausal women. *Menopause* **14**, 293-299.

3. Campbell MJ, Woodside JV, Honour JW *et al.* (2004) Effect of red clover-derived isoflavone supplementation on insulin-like growth factor, lipid and antioxidant status in healthy female volunteers: a pilot study. *Eur J Clin Nutr* **58**, 173-179.

4. Clerici C, Setchell KD, Battezzati PM *et al.* (2007) Pasta naturally enriched with isoflavone aglycons from soy germ reduces serum lipids and improves markers of cardiovascular risk. *The Journal of nutrition* **137**, 2270-2278.

5. Curtis PJ, Potter J, Kroon PA *et al.* (2013) Vascular function and atherosclerosis progression after 1 y of flavonoid intake in statin-treated postmenopausal women with type 2 diabetes: a double-blind randomized controlled trial. *Am J Clin Nutr* **97**, 936-942.

6. Gallagher JC, Satpathy R, Rafferty K *et al.* (2004) The effect of soy protein isolate on bone metabolism. *Menopause* **11**, 290-298.

7. Gardner CD, Messina M, Kiazand A *et al.* (2007) Effect of two types of soy milk and dairy milk on plasma lipids in hypercholesterolemic adults: a randomized trial. *J Am Coll Nutr* **26**, 669-677.

8. Greany KA, Nettleton JA, Wangen KE *et al.* (2004) Probiotic consumption does not enhance the cholesterol-lowering effect of soy in postmenopausal women. *J Nutr* **134**, 3277-3283.

9. Greany KA, Nettleton JA, Wangen KE *et al.* (2008) Consumption of isoflavone-rich soy protein does not alter homocysteine or markers of inflammation in postmenopausal women. *Eur J Clin Nutr* **62**, 1419-1425.

10. Hall WL, Vafeiadou K, Hallund J *et al.* (2005) Soy-isoflavone-enriched foods and inflammatory biomarkers of cardiovascular disease risk in postmenopausal women: interactions with genotype and equol production. *Am J Clin Nutr* **82**, 1260-1268; quiz 1365-1266.

11. Hall WL, Vafeiadou K, Hallund J *et al.* (2006) Soy-isoflavone-enriched foods and markers of lipid and glucose metabolism in postmenopausal women: interactions with genotype and equol production. *The American journal of clinical nutrition* **83**, 592-600.

12. Hallund J, Bugel S, Tholstrup T *et al.* (2006) Soya isoflavone-enriched cereal bars affect markers of endothelial function in postmenopausal women. *Br J Nutr* **95**, 1120-1126.

13. Hodis HN, Mack WJ, Kono N *et al.* (2011) Isoflavone soy protein supplementation and atherosclerosis progression in healthy postmenopausal women: a randomized controlled trial. *Stroke; a journal of cerebral circulation* **42**, 3168-3175.

14. Kreijkamp-Kaspers S, Kok L, Bots ML *et al.* (2005) Randomized controlled trial of the effects of soy protein containing isoflavones on vascular function in postmenopausal women. *Am J Clin Nutr* **81**, 189-195.

15. Kreijkamp-Kaspers S, Kok L, Grobbee DE *et al.* (2004) Effect of soy protein containing isoflavones on cognitive function, bone mineral density, and plasma lipids in postmenopausal women: a randomized controlled trial. *JAMA : the journal of the American Medical Association* **292**, 65-74.

16. Liu ZM, Ho SC, Chen YM *et al.* (2014) Whole soy, but not purified daidzein, had a favorable effect on improvement of cardiovascular risks: a 6-month randomized, double-blind, and placebo-controlled trial in equol-producing postmenopausal women. *Mol Nutr Food Res* **58**, 709-717.

17. Liu ZM, Ho SC, Chen YM *et al.* (2015) Effect of whole soy and purified daidzein on ambulatory blood pressure and endothelial function-a 6-month double-blind, randomized controlled trial among Chinese postmenopausal women with prehypertension. *European journal of clinical nutrition*.

18. Liu ZM, Ho SC, Chen YM *et al.* (2013) A six-month randomized controlled trial of whole soy and isoflavones daidzein on body composition in equol-producing postmenopausal women with prehypertension. *Journal of Obesity* **2013**.

19. Ma Y, Chiriboga D, Olendzki BC *et al.* (2005) Effect of soy protein containing isoflavones on blood lipids in moderately hypercholesterolemic adults: a randomized controlled trial. *J Am Coll Nutr* **24**, 275-285.

20. Mangano KM, Hutchins-Wiese HL, Kenny AM *et al.* (2013) Soy proteins and isoflavones reduce interleukin-6 but not serum lipids in older women: a randomized controlled trial. *Nutr Res* **33**, 1026-1033.

21. McVeigh BL, Dillingham BL, Lampe JW *et al.* (2006) Effect of soy protein varying in isoflavone content on serum lipids in healthy young men. *Am J Clin Nutr* **83**, 244-251.

22. Meyer BJ, Larkin TA, Owen AJ *et al.* (2004) Limited lipid-lowering effects of regular consumption of whole soybean foods. *Ann Nutr Metab* **48**, 67-78.

23. Nestel P, Cehun M, Chronopoulos A *et al.* (2004) A biochanin-enriched isoflavone from red clover lowers LDL cholesterol in men. *Eur J Clin Nutr* **58**, 403-408.

24. Nikander E, Tiitinen A, Laitinen K *et al.* (2004) Effects of isolated isoflavonoids on lipids, lipoproteins, insulin sensitivity, and ghrelin in postmenopausal women. *J Clin Endocrinol Metab* **89**, 3567-3572.

25. Pipe EA, Gobert CP, Capes SE *et al.* (2009) Soy protein reduces serum LDL cholesterol and the LDL cholesterol:HDL cholesterol and apolipoprotein B:apolipoprotein A-I ratios in adults with type 2 diabetes. *J Nutr* **139**, 1700-1706.

26. Pop EA, Fischer LM, Coan AD *et al.* (2008) Effects of a high daily dose of soy isoflavones on DNA damage, apoptosis, and estrogenic outcomes in healthy postmenopausal women: a phase I clinical trial. *Menopause* **15**, 684-692.

27. Pusparini, Yenny, Hidayat A (2015) Effect of soy isoflavone supplementation on endothelial dysfunction and oxidative stress in equol-producing postmenopausal women. *Endocrine, metabolic & immune disorders drug targets* **15**, 71-79.

28. Qin Y, Shu F, Zeng Y *et al.* (2014) Daidzein supplementation decreases serum triglyceride and uric acid concentrations in hypercholesterolemic adults with the effect on triglycerides being greater in those with the GA compared with the GG genotype of ESR-beta RsaI. *J Nutr* **144**, 49-54.

29. Reimann M, Dierkes J, Carlsohn A *et al.* (2006) Consumption of soy isoflavones does not affect plasma total homocysteine or asymmetric dimethylarginine concentrations in healthy postmenopausal women. *The Journal of nutrition* **136**, 100-105.

30. Reverri EJ, LaSalle CD, Franke AA *et al.* (2015) Soy provides modest benefits on endothelial function without affecting inflammatory biomarkers in adults at cardiometabolic risk. *Molecular nutrition & food research* **59**, 323-333.

31. Sen C, Morimoto Y, Heak S *et al.* (2012) Soy foods and urinary isoprostanes: results from a randomized study in premenopausal women. *Food & function* **3**, 517-521.

32. Steinberg FM, Guthrie NL, Villablanca AC *et al.* (2003) Soy protein with isoflavones has favorable effects on endothelial function that are independent of lipid and antioxidant effects in healthy postmenopausal women. *The American journal of clinical nutrition* **78**, 123-130.

33. Thorp AA, Howe PR, Mori TA *et al.* (2008) Soy food consumption does not lower LDL cholesterol in either equol or nonequol producers. *The American journal of clinical nutrition* **88**, 298-304.

34. Tormala R, Appt S, Clarkson TB *et al.* (2008) Equol production capability is associated with favorable vascular function in postmenopausal women using tibolone; no effect with soy supplementation. *Atherosclerosis* **198**, 174-178.

35. Tormala R, Appt S, Clarkson TB *et al.* (2008) Impact of soy supplementation on sex steroids and vascular inflammation markers in postmenopausal women using tibolone: role of equol production capability. *Climacteric* **11**, 409-415.

36. Tormala RM, Appt S, Clarkson TB *et al.* (2007) Individual differences in equol production capability modulate blood pressure in tibolone-treated postmenopausal women: lack of effect of soy supplementation. *Climacteric : the journal of the International Menopause Society* **10**, 471-479.

37. Tormala RM, Nikander E, Tiitinen A *et al.* (2006) Serum cholesterol efflux potential in postmenopausal women treated with isolated isoflavones. *Menopause* **13**, 96-101.

38. van der Velpen V, Geelen A, Hollman PC *et al.* (2014) Isoflavone supplement composition and equol producer status affect gene expression in adipose tissue: a double-blind, randomized, placebo-controlled crossover trial in postmenopausal women. *The American journal of clinical nutrition* **100**, 1269-1277.

39. van der Velpen V, Geelen A, Schouten EG *et al.* (2013) Estrogen receptor-mediated effects of isoflavone supplementation were not observed in whole-genome gene expression profiles of peripheral blood mononuclear cells in postmenopausal, equol-producing women. *J Nutr* **143**, 774-780.

40. Welty FK, Lee KS, Lew NS *et al.* (2007) Effect of soy nuts on blood pressure and lipid levels in hypertensive, prehypertensive, and normotensive postmenopausal women. *Archives of internal medicine* **167**, 1060-1067.

41. West SG, Hilpert KF, Juturu V *et al.* (2005) Effects of including soy protein in a blood cholesterol-lowering diet on markers of cardiac risk in men and in postmenopausal women with and without hormone replacement therapy. *Journal of women's health (2002)* **14**, 253-262.

42. Wong JM, Kendall CW, Marchie A *et al.* (2012) Equol status and blood lipid profile in hyperlipidemia after consumption of diets containing soy foods. *Am J Clin Nutr* **95**, 564-571.
